# Effects of the COVID-19 pandemic on working conditions of maternity staff – a scoping review

**DOI:** 10.1186/s12884-025-07905-5

**Published:** 2025-08-14

**Authors:** Marcus Heise, Murielle Madi, Elke Mattern, Antonia Stengler, Anke Steckelberg

**Affiliations:** 1 Institute of Health and Nursing Science, Medical Faculty, Martin Luther University Halle-Wittenberg, Magdeburger Str. 8, Halle (Saale), 06112 Germany; 2 University Clinic and Policlinic for Visceral, Vascular and Endocrine Surgery, Ernst-Grube-Straße 40, Halle (Saale), 06120 Germany

**Keywords:** Maternity care, Obstetrician, Midwife, Maternal Health Services, COVID‑19, Occupational Stress

## Abstract

**Background:**

The COVID-19 pandemic significantly disrupted healthcare systems, with a pronounced impact on maternity care. Midwives and obstetricians faced numerous structural, organizational, and subjective challenges in maintaining high-quality care under unprecedented conditions. This review examines the multifaceted effects of the COVID-19 pandemic on maternity staff and the challenges encountered during this period.

**Methods:**

This scoping review adhered to the methodologies outlined by Arksey & O'Malley and the Joanna Briggs Institute. We searched six bibliographic databases for English and German articles published between January 2020 and September 2023 that addressed the pandemic's impact on maternity staff in OECD countries. The themes and subthemes were deductively established from the extracted results, synthesized into descriptive narratives and charted within a schematic diagram. The reporting followed the PRISMA-ScR statement.

**Results:**

This scoping review included 83 articles. Key findings were categorized into the two broader topics “structural challenges” and “mental health impacts on the workforce”. Structural challenges included staff shortages, restructuring, inadequate personal protective equipment (PPE), transition to virtual communication, managing SARS-CoV-2 positive patients, and restrictions on accompanying persons. Mental health impacts were significant, with increased levels of anxiety, stress and moral dilemmas among staff. Despite these challenges, a strong sense of occupational solidarity was observed.

**Conclusions:**

The findings emphasize the need for improved support systems for maternity care staff during pandemics to mitigate these adverse effects. Recommendations include better resource allocation, enhanced mental health support, and clear communication strategies to navigate future healthcare crises effectively. These results may inform pandemic preparedness for future health crises.

**Trial Registrations:**

This scoping review was registered with OSF on October 24th, 2023 and the published protocol is openly available via https://doi.org/10.17605/OSF.IO/AVYDX

**Supplementary Information:**

The online version contains supplementary material available at 10.1186/s12884-025-07905-5.

## Background

The COVID-19 pandemic has profoundly impacted healthcare systems worldwide, presenting unprecedented challenges to healthcare providers [1, 2]. This strain was particularly evident in maternity care, where midwives and obstetricians faced numerous structural, organizational, and subjective challenges in ensuring the safety and well-being of both mothers and newborns [3–5]. Maternity care is unique in that it cannot be paused or postponed, unlike elective surgeries, as pregnancies and births continue regardless of external crises [6, 7]. This essential service required a delicate balance between effective infection control measures and the continued provision of care, making adaptations during the COVID-19 pandemic particularly challenging [8, 9].

The landscape of maternal healthcare provision during the COVID-19 pandemic was in particular complicated by organizational challenges, including staff shortages, workforce restructuring, insufficient personal protective equipment, the transition to virtual communication, and the exclusion of accompanying persons [10–14]. Healthcare systems worldwide responded to these challenges in various ways, leading maternity staff and healthcare institutions to implement different strategies to adapt and ensure the continued provision of high-quality care. The impact of COVID-19 led to transformative changes in maternity healthcare, including the introduction of remote antenatal and postnatal appointments, the redeployment of midwives to different sectors, and a significant reduction in postnatal care [15–17]. The reduction in services led to decreased access to essential maternity care, resulting in compromised outcomes for mothers and newborns. Many routine in-person visits were replaced by virtual consultations, which, while beneficial in reducing exposure, sometimes fell short in providing comprehensive care. The exclusion of partners and support persons during labor also negatively impacted the birthing experiences and emotional supports for mothers [9, 18].

These structural and organizational challenges were accompanied by subjective effects on maternity staff, including mental strain, anxiety, and depression. The emotional toll of dealing with mothers who tested positive for SARS-CoV-2, maintaining maternal healthcare provision in the face of uncertainty, the fear of contracting or transmitting the virus, and the fear of not providing adequate care to patients added additional layers of complexity to the experiences of maternity professionals [19–21].

Understanding the depth and consequences of these challenges and strategies is crucial for informing future public health crises and optimizing maternal and newborn health outcomes. Two significant scoping reviews have already provided important insights on this topic. Flaherty et al. [10] examined the experiences of women and maternity care providers during the COVID-19 pandemic, while Schmitt et al. [5] focused on the effects of the pandemic on maternity staff in 2020. Our review builds upon and extends these works in several important aspects. First, we expand the timeframe to September 2023, capturing long-term impacts and adaptations in maternity care. Second, we place a particular emphasis on structural challenges and mental health of staff, allowing for a deeper understanding of systemic changes. Third, we integrate both qualitative and quantitative studies to paint a more comprehensive picture of maternity staff experiences. The aim of our scoping review is to synthesize the existing body of knowledge on the challenges faced by maternity staff during the delivery of care amid the COVID-19 pandemic. By exploring both structural and organizational challenges and subjective effects, we aim to provide a comprehensive overview of the multifaceted issues encountered by maternity professionals. Furthermore, our review aims to identify and characterize the strategies employed by maternity staff and healthcare institutions to address these challenges. Thus, we seek to contribute insights that may guide the development of strategies for future public health crises.

## Methods

To ensure transparency in conducting this scoping review, we followed the five-step framework proposed by Arksey and O'Malley [22] and the Joanna Briggs Institute’s Manual for Evidence Synthesis [23]. The reporting followed the PRISMA statement (Preferred Reporting Items for Systematic Reviews and Meta-analysis Protocols) [24, 25]. The corresponding PRISMA Extension for Scoping Reviews Reporting Statement (PRISMA-ScR) [26] is provided in Additional file 1.

This scoping review was registered with OSF on October 24th, 2023 and the published protocol is openly available via https://doi.org/10.17605/OSF.IO/AVYDX

### Formulating the research question

Until September 2023, what publications have been published on the experiences of maternity staff in OECD countries regarding the challenges they faced in providing care during the COVID-19 pandemic, and what strategies have been implemented to address the impact of the COVID-19 pandemic?

### Identifying relevant studies

From 1 st of August 2023 to 25th of September 2023, published articles were identified by searching the following six databases: MEDLINE via PubMed, Web of Science Collection via Clarivate, CINAHL via EBSCO, the Cochrane Library, PSYNDEX and APA PsychNet. Titles and abstracts were searched, based on an adapted search string used by Schmitt et al. [5]. To enhance the original search strategy, supplementary search terms were incorporated to include additional professional health care providers and various aspects of their working conditions during the COVID-19 pandemic. The search string was iteratively refined and discussed among the authors until a consensus was reached. Additional file 2 details the search strings used for each database.

To identify additional publications, we manually screened the following two journals, applying the same criteria for publication date and language as used in the database search: Journal of Midwifery Science (Zeitschrift für Hebammenwissenschaft) of the German Society of Midwifery Science and The Midwife (Die Hebamme).

### Study selection

The scoping review included systematic reviews, primary studies (both qualitative and quantitative in design), experience reports, case series and position statements with quantifiable evidence that addressed challenges, barriers and facilitators in the provision of maternal health care during the COVID-19 pandemic. Study protocols were not included.

Solely evidence that addressed the subjective perspectives and experiences of maternity staff was eligible for inclusion. Our definition of maternity staff included midwives, obstetricians, obstetric nurses, and nurse-midwives. Studies in which the target population solely consisted of students, mothers, newborns, relatives of patients, doulas, trainees, resident physicians or physician-applicants were not eligible. Studies with heterogeneous populations were included only if the results for the maternity staff were reported separately as a distinct subgroup.

Evidence relating to the training of obstetricians or general research in obstetrics was only included if it focused on the perspective of professional maternity staff. Studies that did not explicitly address the COVID-19 pandemic in the context of maternity care and instead contained general guidelines for practice were excluded. Further, studies related to obstetrical anesthesia were also not eligible. To reduce the heterogeneity of the health care systems investigated in this review, solely populations belonging to the Organization for Economic Co-operation and Development (OECD) were eligible. Studies that examined (at least one) non-OECD countries were excluded. The decision to restrict the review to OECD countries stemmed from the need to reduce systemic heterogeneity in healthcare infrastructures, regulatory frameworks, and resource allocation. OECD nations share comparably standardized maternal care systems, enabling clearer cross-country analysis of pandemic-related adaptations. For instance, the widespread adoption of telehealth in countries like Germany or France contrasted with fragmented implementations in non-OECD settings, where internet access and digital literacy vary widely [27]. For qualitative studies and case series, a sample size of n > 5 was a prerequisite for inclusion. The publication date was limited to the period between 1 st of January 2020 and 26th of September 2023. Studies must have been published in English or German.

The study selection process consisted of two primary phases: initial title and abstract (TiAb) screening, followed by full text (FT) screening, both based on the predefined inclusion criteria described above. In each phase, two reviewers (EK and MH) independently assessed the titles and abstracts of the retrieved articles. Disagreements were discussed with a third reviewer (MM), until consensus was reached. In accordance with standard guidance on the conduct of scoping reviews [22, 23], neither the methodological quality nor risk of bias of the included articles were assessed. Records containing only a title without accompanying text were still included in the subsequent FT phase, skipping the TiAb screening. In cases where it was unclear from the abstract whether two records were duplicates, a similar procedure was applied. These meticulous screening procedures ensured a systematic and comprehensive selection of studies for inclusion in the scoping review.

Screening was conducted using the Rayyan platform [28].

### Charting the data

Included studies were documented using a PRISMA 2020 Flow Diagram, and an overview of these studies was provided in table format.

To prepare the descriptive summary, Arksey and O’Malley’s analytical framework [22] was adapted. For topical analysis, an additional data extraction sheet was iteratively developed and piloted. Data were extracted by one author (MH) and independently checked for precision by two co-authors (MM and EM). Disagreements were solved by discussion. The following data were extracted: a) endpoints (concepts/inventories), b) predictors/influencing variables, c) relevant associations and effect measures, d) structural and organizational challenges to the provision of care during COVID-19, e) subjective effects on maternity staff, f) further barriers to the provision of standard care, g) facilitators of standard care, h) changes in care due to COVID-19, i) additional important results and topics regarding the working conditions of maternity staff during COVID-19, j) authors'conclusions, k) recommendations for future pandemics, l) limitations and robustness of conclusions, m) plausibility of conclusions and consistency with reported results, n) extractor's own remarks and comments.

### Summarizing, and reporting the findings

Based on the data extraction, one author (MH) organized the literature by categorizing the included studies into themes. The emerging themes, formed through an iterative process of deductive assumptions derived from the previous reviews [5, 10] and inductive adjustments, reflected the impact of the COVID-19 pandemic on maternity staff. Corresponding themes and subthemes were deductively identified and the extracted results of the included studies were assigned accordingly.

The results were then synthesized and descriptions of the themes and their subthemes were formulated. All authors reviewed each description for clarity and readability, with collaborative efforts to edit the descriptions for accuracy. The results were then reported narratively, with a comprehensive summary of the topics presented in a schematic diagram to illustrate the relationships between them.

## Results

### Overview of included studies

The database search yielded 2,611 records published between January 2020 and September 2023, with 1,125 duplicates removed prior to screening. During the TiAb screening phase, 286 publications were identified for full-text retrieval. In cases where full texts were not readily available, authors were contacted both via email and ResearchGate, resulting in seven additional publications. However, one article could not be obtained despite these attempts.

**Fig. 1:**
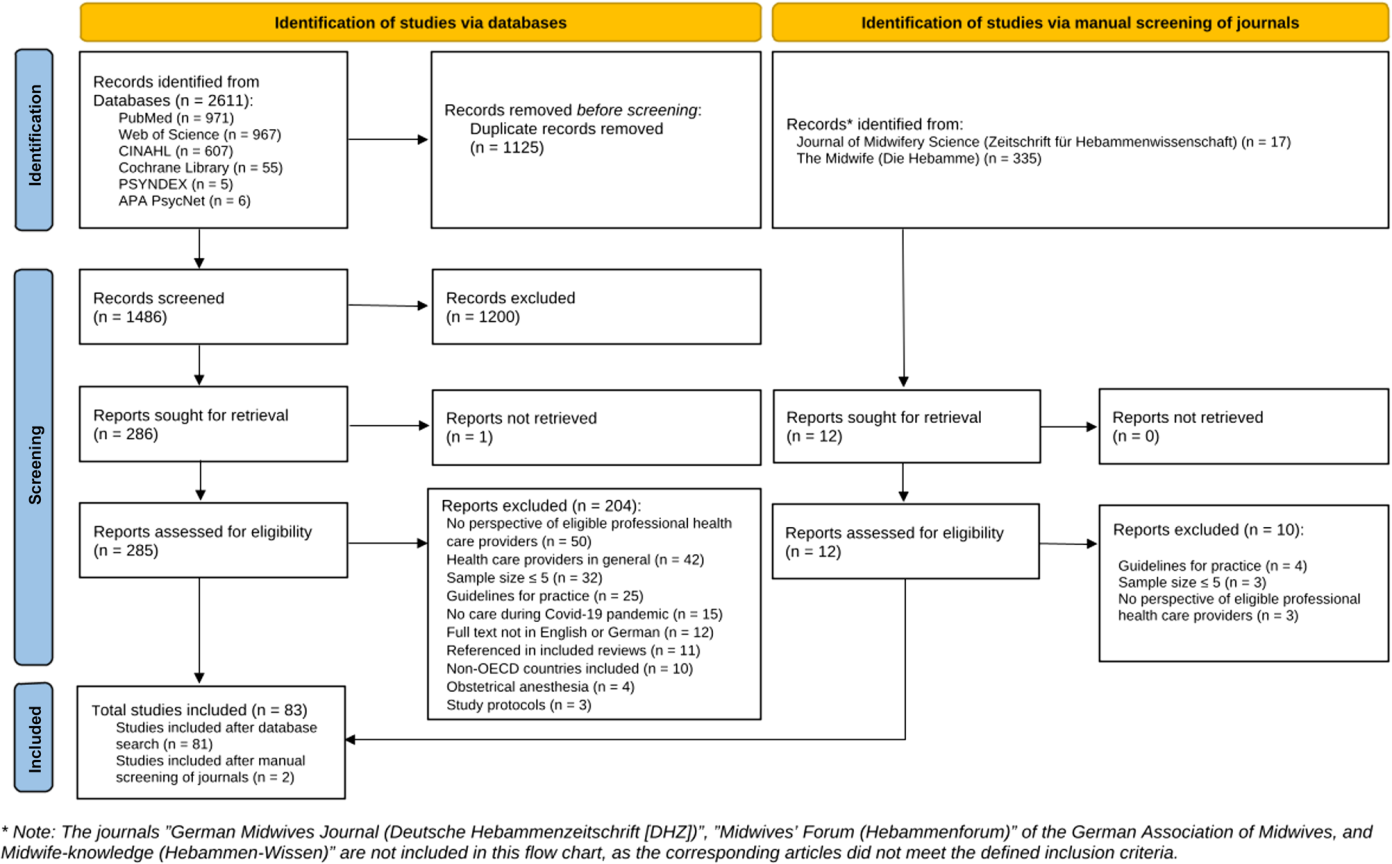
Prisma 2020 Flow Diagram. * The journals”German Midwives Journal (Deutsche Hebammenzeitschrift [DHZ])”,”Midwives’ Forum (Hebammenforum)” of the German Association of Midwives, and Midwife-knowledge (Hebammen-Wissen)” are not included in this flow chart, as the corresponding articles did not meet the defined inclusion criteria

Within the FT screening phase, 204 reports were excluded. Of note, eleven studies that were already referenced within included reviews [5, 10] were excluded from this FT screening as their results were considered redundant (see additional file 3). The review included 81 studies from the database search, with two additional studies added after TiAb and FT screening of manually screened journals, bringing the total to 83 studies, as shown in Fig. 1.

The majority of studies originated from USA (*n* = 19), followed by UK (*n* = 13), Australia (*n* = 9) Turkey (*n* = 7), Germany (*n* = 4), Italy (*n* = 4), Netherlands (*n* = 4), Israel (*n* = 3), Switzerland (*n* = 3), Canada (*n* = 2), France (*n* = 2), Ireland (*n* = 2), Japan (*n* = 2), Poland (*n* = 2), Spain (*n* = 2), New Zealand (*n* = 1) and Norway (*n* = 1). Three studies were multinational.

Of the 83 studies included, 52 were quantitative studies, 21 were qualitative studies and 10 were mixed methods studies. Of note, only 2 randomized controlled trials were included. Of the 83 included studies, two publications were not peer-reviewed and contained qualitative-anecdotal descriptions of an intervention.

An overview of the included studies is provided in Table 1.

**Table 1:** Overview of the included studies

Author, Year	Title	Type of Article	Topic	Country of focus	Included Participants/ studies	Conclusion
Akselsen et al., 2023 [29]	Midwives'experiences with providing home-based postpartum care during the COVID-19 pandemic: A qualitative study	qualitative study (semi-structured individual interviews) peer reviewed	midwives'experiences with providing homebased postpartum care during the COVID-19 pandemic	Norway	n = 11 midwives experienced in offering homebased postpartum care	Results highlight face-to-face postpartum care's importance but recognize telehealth as useful in limited-access situations
Appelman et al., 2022 [30]	It was tough, but necessary. Organizational changes in a community based maternity care system during the first wave of the COVID-19 pandemic: A qualitative analysis in the Netherlands	qualitative study (semi-structured individual interviews) peer reviewed	maternity care providers’ opinions on the changes in maternity care during the COVID-19 pandemic	Netherlands	n = 6 obstetricians, n = 7 community-based midwives, n = 2 hospital-based midwives, n = 2 obstetric nurses	Participants emphasized maintaining routine intrapartum care, urging maternity staff retention over redeployment to COVID-19 units. Participants suggested enhancing telehealth, and prioritizing provider safety for sustainability
Arnold et al., 2024 [19]	"I might have cried in the changing room, but I still went to work". Maternity staff balancing roles, responsibilities, and emotions of work and home during COVID-19: An appreciative inquiry	qualitative study (semi-structured individual interviews and group discussions) peer reviewed	wellbeing and individual strategies of maternity staff in balancing professional and personal challenges during the COVID-19 pandemic	UK	n = 39 maternity staff (maternity support workers, student midwives, newly qualified and experienced midwives)	The findings underscore the complex, interrelated issues faced by maternity staff during COVID, as they had to balance professional obligations while also being invested in their multiple roles outside of work. Maternity staff found meaning in the values underpinning their work
Blauer et al.,2021 [31]	Leadership in Zeiten von Corona: Die Perspektive von Pflegenden und Hebammen in einem Universitätsspital [Leadership in times of corona: the perspective of nurses and midwives in a university hospital]	quantitative, cross-sectional study (action research) peer reviewed	evaluation of leadership in a Swiss university hospital during the pandemic	Switzerland	n = 57 maternity staff (maternity ward, antenatal ward, mother and child ward)	Respondents felt heavily burdened by personal circumstances, changing directives, information overload, and constant workflow adjustments. In obstetrics, the absence of federal health authority guidelines for pregnant individuals led to additional uncertainties
Boyle et al., 2022 [32]	Perinatal bereavement care during COVID-19 in Australian maternity settings	quantitative, cross-sectional study including thematic analyses of free-text comments peer reviewed	care providers’ perspectives of the impact of COVID-19 on implementing respectful and supportive perinatal bereavement care and practice changes that have ensued	Australia	n = 35 health care providers who provided perinatal bereavement care (clinical settings, support organizations, research)	Implementing best practice perinatal bereavement care during the pandemic presented significant challenges for healthcare providers. Recommendations include adjusting communication methods, advocating for compassionate allowances, enhancing telehealth communication, providing private spaces, connecting with support organizations and establishing COVID-19-specific guidelines
Bradfield et al., 2021 [33]	Experiences of receiving and providing maternity care during the COVID-19 pandemic in Australia: A five-cohort cross-sectional comparison	quantitative, cross-sectional study peer reviewed	experiences of those receiving or providing care during the COVID-19 pandemic	Australia	n = 560 midwives; n = 78 medical practitioners (General Practitioners, Obstetricians, Neonatologists and other medical practitioners)	In the context of maternity care, ensuring the safety of healthcare providers during COVID-19 exposure posed unique challenges, particularly in the hospital setting. Midwives and obstetricians expressed heightened levels of concern about personal risk. Significant changes in the delivery of maternity care, such as the introduction of telemedicine and physical distance during hospital visits, have particularly affected practice dynamics
Brown et al., 2022 [34]	A Qualitative Study Focused on Maternity Care Professionals'Perspectives on the Challenges of Providing Care During the COVID-19 Pandemic	qualitative analysis of free-text responses from a cross-sectional study peer reviewed	maternity care professionals’ perceptions of the impact of COVID-19 on maternity care	USA	n = 647 maternity care professionals in hospital settings (nurses, physicians, and midwives)	Maternity care professionals faced increased stress, fatigue, and uncertainty during COVID-19 and adapted with policy changes and telehealth, but experienced increased anxiety and workload
Buek et al., 2022 [35]	Opportunities and challenges for family-centered postpartum care during the COVID-19 pandemic: a qualitative study of nurse perspectives	qualitative study (structured individual interviews) peer reviewed	nurses'perceptions of COVID-19 policy changes and their impact on postpartum care delivery	USA	n = 20 postpartum nurses from five hospitals	Restricted visitation policies instituted during COVID-19 had positive impact on family-centered care such as improved mother–child bonding, maternal rest, and breastfeeding. Many of the nurses interviewed said they hoped that visitor restrictions for postpartum care would continue after the pandemic
Cooke et al., 2021 [36]	Exploring the STEP-uP to practice: A survey of UK Lead Midwives for Education views of the STudent midwife Extended Practice Placement during the first wave of the COVID-19 pandemic	mixed methods cross-sectional study (descriptive analysis and thematic analysis of free-text responses) peer reviewed	options, barriers and facilitating factors in providing midwifery education during the COVID-19 pandemic; Mobilization of students to support the midwifery workforce	UK	n = 38 lead midwives for education	Extended placements and deployment of students in the NHS workforce were implemented in order to compensate for the increased workload during the COVID-19 pandemic. Clarity is needed to avoid blurring the student's role with that of a healthcare support workers and to enable the completion of educational program requirements
Cronin et al., 2020 [37]	Perceptions of patients and providers regarding restriction of labor and delivery support people in the early stages of the coronavirus disease 2019 pandemic	quantitative, cross-sectional study (research letter) peer reviewed	perceptions of providers regarding restriction of labor and delivery support people in the early stages of the coronavirus disease 2019 pandemic	USA	n = 65 Labor & Delivery team members within a single hospital	A high proportion of providers reported that they were personally very concerned about becoming infected. Although a large proportion were in favor of restricting patient visits, many providers expressed concern about negative effects on the birthing experience
Crowther et al., 2021 [38]	When Maintaining Relationships and Social Connectivity Matter: The Case of New Zealand Midwives and COVID-19	qualitative content analysis of media websites peer reviewed	midwives'issues, challenges, and experiences, as narrated by media reports in New Zealand during the initial surge of the COVID-19 pandemic	New Zealand	n = 71 pertinent articles (media reports on midwives and maternity in New Zealand)	NZ midwives adapted and provided quality care amidst pandemic challenges, prioritizing women's choices and cultural needs even during situations of national crisis. The results indicate a lack of preparedness for a pandemic and inadequate provision of PPE
De Backer et al., 2022 [39]	Precarity and preparedness during the SARS-CoV-2 pandemic: A qualitative service evaluation of maternity healthcare professionals	qualitative study (semi-structured individual interviews) peer reviewed	examine maternity staff experiences of pandemic care in terms of coping with health system pressures and lack of preparedness	UK	n = 29 maternity services staff (midwives, and obstetric staff)	Maternity services faced ongoing strain and lacked preparation for a pandemic crisis. The pandemic caused disruptions in service delivery with lasting consequences, leading to fragmentation of care and systemic shortages of human resources
Del Piccolo et al., 2021 [40]	The Psychological Impact of COVID-19 on Healthcare Providers in Obstetrics: A Cross-Sectional Survey Study	quantitative, cross-sectional study peer reviewed	factors and associations of psychological distress among obstetric health care providers during the COVID-19 Pandemic	Italy	n = 481 Providers in hospital settings (gynecologists, obstetricians, and midwives)	Psychological distress among obstetrics health care providers during COVID-19 necessitates interventions at individual, interpersonal, and organizational levels to promote resilience and well-being. The fear of infecting the family and the continuous updating of recommendations and measures to be implemented were the most perceived distressing factors
Dennehy et al., 2023 [41]	An extra level of kind of torment': Views and experiences of recurrent miscarriage care during the initial phases of COVID-19 in Ireland-A qualitative interview study	qualitative study (semi-structured individual interviews) peer reviewed	impact of the COVID ‐ 19 pandemic on experiences of recurrent miscarriage services and perceptions of care	Ireland	n = 42 service providers involved in the management or delivery of recurrent miscarriage services and supports	During the COVID-19 pandemic, the provision of care for recurrent miscarriages suffered from changes in service provision, service reduction and redeployment. Personal support and partner involvement for comprehensive care were not possible
Deruelle et al., 2021 [42]	Prenatal care providers'perceptions of the SARS-Cov-2 vaccine for themselves and for pregnant women	quantitative, cross-sectional study peer reviewed	perceptions of French prenatal care providers regarding SARS-CoV-2 vaccination during pregnancy	France	n = 1,416 prenatal care providers (obstetricians, gynecologists, midwives, GPs)	Most French prenatal healthcare providers were favorable towards vaccinating pregnant women, but a large minority express reservation. Prenatal care providers play an important role in the acceptance of SARS-Cov-2 vaccination for pregnant women
Dogan et al., 2023 [43]	Compassion fatigue and moral sensitivity in midwives in COVID-19	quantitative, cross-sectional study peer reviewed	Compassion fatigue impact on midwives'moral sensitivity during COVID-19	Turkey	n = 310 midwives	Midwives'compassion fatigue during the pandemic period was high. Secondary trauma and occupational burnout sub-dimensions were significant for compassion fatigue and decreased moral sensitivity. The implementation of psychological support interventions that will reduce the compassion fatigue of midwives should be given priority
Elling et al., 2022 [44]	Women's and Nurses'Perceptions of Visitor Restrictions After Childbirth During the COVID-19 Pandemic	cross-sectional, mixed-methods study peer reviewed	perceptions of labor and delivery nurses in the postpartum period regarding a restricted visitor policy during the COVID-19 pandemic	USA	n = 47 labor and delivery nurses (single hospital)	Nurses supported visitor restrictions because they improved the quality of care and reduced distractions. However, they acknowledged potential disadvantages such as reduced social support and advocated for flexibility to meet mothers'needs
Ellis et al., 2023 [45]	Changes to Birth Plans Due to COVID-19: A Survey of Utah Midwives and Doulas	quantitative, cross-sectional study peer reviewed	experiences of midwives and doulas during the COVID-19 pandemic regarding perceived impact on choice of birth setting and provision of PPE	USA	n = 68 midwives	The COVID-19 pandemic led to changes in community birth practices, resulting in an increase in out-of-hospital births and a shortage of PPE. Midwives expressed concern about providing home care during COVID-19 because not all patients were routinely tested for COVID-19
Erin et al., 2022 [46]	Psychosocial outcomes of COVID-19 pandemic on healthcare workers in maternity services	quantitative, cross-sectional study peer reviewed	effects of the COVID-19 outbreak on social support and anxiety levels in healthcare professionals working in maternity services	Turkey	n = 96 healthcare workers in maternity services in single-hospital setting (midwives, doctors, nurses)	Throughout the COVID-19 pandemic, a significant proportion of healthcare workers experienced decreased social support, leading to increased anxiety. Differences in support among healthcare workers were negligible
Flaherty et al., 2022 [10]	Maternity care during COVID-19: a qualitative evidence synthesis of women's and maternity care providers'views and experiences	qualitative evidence synthesis peer reviewed	experiences of maternity care providers during the COVID-19 Pandemic	international scope	n = 48 studies; population of interest maternity care providers, who provided qualitative data on their experiences of maternity care	The experiences of maternity care providers during COVID-19 were categorized into three themes: altered care, professional/personal impact, and broader structural implications. Findings highlight negative experiences for providers in all three themes. Maternity care altered significantly because of the pandemic
Fumagalli et al., 2023 a [47]	Midwives'experiences of providing maternity care to women and families during the COVID-19 pandemic in Northern Italy	qualitative study (semi-structured individual interviews) peer reviewed	midwives’ stress, anxiety and moral distress working in a specialized referral center for COVID-19 positive women during the pandemic	Italy	n = 15 midwives in antenatal/postnatal clinical setting	Midwives faced professional and personal challenges during the pandemic, displaying feelings of fear, anxiety, uncertainty, discomfort, lack of support and knowledge with potential long-term effects. Adjusting to the continuous, rapid and drastic re-organization of maternity services was particularly challenging
Fumagalli et al., 2023 b [48]	Volunteering in an emergency project in response to the COVID-19 pandemic crisis: the experience of Italian midwives	qualitative study (semi-structured individual interviews and written diaries) peer reviewed	experiences of midwives who volunteered on a regional helpline, providing advice on health issues or access to ambulance services	Italy	n = 59 midwives volunteering in the emergency phone service	Midwives in volunteer activities noted significant impacts on both professional and personal aspects of their lives. Volunteer experiences were generally positive, challenging and enriching. However, midwives also faced unexpected physical and emotional fatigue, exacerbated by the severity of the pandemic, which caused emotional distress
Gamberini et al., 2023 [49]	Effect of COVID-19 on antenatal care: experiences of medical professionals in the Netherlands	qualitative study (semi-structured individual interviews and document analysis of guidelines) peer reviewed	impact of COVID-19 guidelines on antenatal care provision in the Netherlands and its effects on midwives and gynecologists	Netherlands	n = 9 gynecologists and n = 11 midwives in community practices or hospitals; n = 9 guidelines	The COVID-19 pandemic led to significant changes in antenatal care protocols, resulting in logistical challenges and delays. Concerns arose about the shift to home monitoring, highlighting the importance of face-to-face care. Midwives faced equipment shortages and increased workloads, exacerbated by the redeployment of staff to COVID-19 care, which affected the provision of standard antenatal care
Gaucher et al., 2022 [50]	The challenge of care coordination by midwives during the COVID-19 pandemic: a national descriptive survey	quantitative, cross-sectional study peer reviewed	midwives'experiences of referral and collaboration with hospitals in the early stages of the pandemic	France	n = 1491 independent midwives (community healthcare)	The pandemic has exacerbated care challenges in France. 64.7% of respondents reported increased difficulties with referrals, 71.0% with hospital collaboration. Nearly half noted potential harm from delayed care
Gemperle et al., 2022 [51]	Midwives'perception of advantages of health care at a distance during the COVID-19 pandemic in Switzerland	quantitative, cross-sectional study peer reviewed	midwives’ perceptions of the advantages of telemedicine during the COVID-19 pandemic	Switzerland	n = 630 midwives	The majority of midwives reported negative experiences with telemedicine. Only 24.1% explicitly mentioned pandemic-related benefits such as"protection from COVID-19"and"continuity of care"and wished to use telehealth beyond the pandemic
George et al., 2021 [52]	Roles and Experiences of Registered Nurses on Labor and Delivery Units in the United States During the COVID-19 Pandemic	quantitative, cross-sectional study (including content analysis of open-ended responses) peer reviewed	perceived changes in the roles and responsibilities of labor and delivery nurses during the COVID-19 pandemic	USA	n = 757 labor and delivery nurses (hospital-setting)	The COVID-19 pandemic led to significant changes in nurses'roles and responsibilities, with nearly half reporting additional duties such as phlebotomy and cleaning. These adjustments, including shifts to unfamiliar units, affected nurses'ability to provide direct care
Giusti et al., 2022 [53]	Prevalence of breastfeeding and birth practices during the first wave of the COVID-19 pandemic within the Italian Baby-Friendly Hospital network. What have we learned?	quantitative, cross-sectional study peer reviewed	evaluation of the Baby-Friendly Hospital Network for adherence to recommended practices, such as exclusive breastfeeding, presence of a companion of the mother's choice, skin-to-skin and rooming-in	Italy	n = 68 participating hospitals	The Baby-Friendly Hospital Network model has improved hospital preparedness. Challenges included increased interpersonal distance, absence of birth attendants, and inadequate professional support, which affect breastfeeding outcomes and maternal satisfaction. Mothers experienced isolation during COVID-19
Goberna-Tricas et al., 2021 [54]	The COVID-19 Pandemic in Spain: Experiences of Midwives on the Healthcare Frontline	qualitative study (structured individual interviews) peer reviewed	the emotional and psychological components of working conditions during COVID-19 among midwives on the frontline of care	Spain	n = 10 midwives (hospital and independent care)	Midwives faced fear, uncertainty, and loneliness in the midst of COVID-19. Lack of protective equipment and conflicting information heightened anxiety. The pandemic exacerbated challenges to decision-making and women's empowerment, dehumanizing the relationship between birth and health care
Goldstein et al., 2022 [6]	Impact of COVID-19 on perinatal care: Perceptions of family physicians in the United States	cross-sectional, mixed-methods study peer reviewed	impact of the COVID- 19 pandemic on perinatal health care delivery from the perspective of family physicians	USA	n = 1055 family physicians providing perinatal care	Family physicians highlighted the impact of COVID-19 on the patient experience, including visitation policies, PPE use, and testing. Perceived impacts on perinatal care included visitation restrictions, PPE challenges, changes in care delivery, and disruptions in continuity of care, with concerns about family separation and disparities
Gotlib et al., 2021 [55]	Is job seniority a protective factor against anxiety among midwives during the SARS-CoV-2 pandemic?	quantitative, cross-sectional study peer reviewed	analyses of the influence of professional experience on the level of anxiety (GAD-7) in a group of midwives during the SARS-CoV-2 pandemic	Poland	n = 100 midwives	Professional experience did not correlate with anxiety levels. Anxiety varied among midwives: no anxiety (20%), mild (35%), moderate (30%) and severe (15%). Fear of COVID-19 and working in primary referral hospitals were associated with higher levels of anxiety
Halperin et al., 2022 [56]	Exploring midwives'coping and functioning in the labour wards during the COVID-19 pandemic from the Labour Ward Head Nurses'perspective: A qualitative study	qualitative study (structured individual interviews) peer reviewed	midwives'coping and functioning in labor wards during the COVID-19 pandemic from the perspective of labor ward head nurses	Israel	n = 13 labor ward head nurses	During the first lockdown in Israel, midwives faced stress, anxiety, and frustration due to fluctuating staffing, lack of PPE, and evolving guidelines. Psychological stress resulted from uncertainty about the duration of the crisis and the lack of therapies or vaccines. Mitigating factors included adequate protection, cooperation, and a sense of duty that fostered high morale
Hanley et al., 2022 [57]	Implementation of Public Health England infection prevention and control guidance in maternity units in response to the COVID-19 pandemic	qualitative study (structured individual interviews) peer reviewed	successes and barriers to implementing Public Health England (PHE) infection prevention and control guidance in English maternity units during the COVID-19 pandemic	UK	n = 16 obstetricians, midwives and neonatologists	Successes in implementing Public Health England's infection prevention and control guidelines were attributed to infrastructure, training satisfaction, and a multidisciplinary approach; barriers included conflicting guidelines, PPE shortages, and testing variations
Henry et al., 2022 [58]	Effects of the COVID-19 Pandemic and Telehealth on Antenatal Screening and Services, Including for Mental Health and Domestic Violence: An Australian Mixed-Methods Study	cross-sectional, mixed-methods study (survey & structured individual interviews) peer reviewed	impact of COVID-19 on domestic and family violence and mental health screening and service delivery in Australian antenatal care in the context of enhanced telehealth support	Australia	staff directly involved in pregnancy care (doctors, midwives, and allied health): n = 17 interviews; n = 109 survey responses	More than half of respondents felt that COVID-19 negatively affected prenatal care, including domestic and family violence and mental health screening. Telehealth offered convenience but posed challenges, especially for high-risk cases. Woman-centered care was compromised
Hertle et al., 2022 a [59]	Digital midwifery care in the pandemic: rapid implementation and good acceptance	quantitative, cross-sectional study peer reviewed	evaluation of newly introduced digital midwifery services during the COVID-19 pandemic in Germany	Germany	n = 1551 midwives	Half of the midwives surveyed provided digital services and wanted to continue after the pandemic. Not all services were considered equally suitable for digital delivery, with midwives valuing infection prevention and time savings
Hertle et al., 2022 b [60]	Midwives'and women's views on digital midwifery care in Germany: Results from an online survey	quantitative, cross-sectional study peer reviewed	opportunities and challenges of digital services including future perspectives after the COVID-19 pandemic from the perspective of midwives	Germany	n = 1551 midwives	The midwives surveyed largely saw digital care as a useful addition to, but not a replacement for, in-person visits. Services that lent themselves to digital adoption were education and counseling, while challenges were noted with physical exams. Midwives cited issues with privacy and reimbursement
Hijdra et al., 2022 [61]	Experiences of Dutch Midwives Regarding the Quality of Care during the COVID-19 Pandemic	qualitative study (structured individual interviews) peer reviewed	quality of care during the COVID-19 pandemic from the perspective of Dutch midwives in primary care	Netherlands	n = 15 midwives	Midwives struggled with challenges such as maintaining quality of care, clarity of information, lack of PPE, and increased workload during COVID-19. Telemedicine created communication barriers. Shared decision making declined amid guideline compliance
Holman et al., 2023 [62]	Telehealth Adoption During COVID-19: Lessons Learned from Obstetric Providers in the Rocky Mountain West	qualitative study (structured individual interviews) peer reviewed	examining obstetric providers'adaptation of telemedicine in rural communities and implications for its institutionalization	USA	n = 20 obstetric providers (obstetricians, gynecologists, maternal–fetal medicine specialists and certified nurse-midwives)	Obstetric providers found telemedicine to be beneficial for antenatal and postpartum care and intended to continue using it after the pandemic. Concerns about equitable access, patient engagement, digital literacy, and infrastructure challenges were reported, highlighting the need for support and training
Iobst et al., 2023 [63]	Challenges, Job Satisfiers, and Self-Care among Perinatal Nurses in the United States during the COVID-19 Pandemic	quantitative, cross-sectional study peer reviewed	perceived challenges, job satisfiers, and self-care of perinatal nurses in the United States during the COVID-19 pandemic	USA	n = 297 perinatal nurses	Challenges faced by healthcare providers during the pandemic included changing policies, shortages of personal protective equipment, and visitor restrictions. Job satisfaction stemmed from providing quality care despite reported overtime and resource constraints. Self-care strategies were prevalent, but barriers such as PPE hindered communication and fear of infection persisted. Administrative decisions and poor compliance contributed to stress
Jacobsen et al., 2022 [64]	Midwifery in the Time of COVID-19: An Exploratory Study from the Perspectives of Community Midwives	qualitative study (structured individual interviews) peer reviewed	challenges, achievements and needs of community midwifery during the COVID-19 pandemic	USA	n = 11 midwives	The results revealed challenges faced by community midwives during COVID-19, including lack of PPE, stigma, and strained relationships with hospitals. Increased demand and uncompensated work contributed to burnout. Telemedicine offered both opportunities and obstacles to ensuring equal access to healthcare for all patients
Jasinski et al., 2021 [65]	Workload, job satisfaction and occupational stress in polish midwives before and during the COVID-19 pandemic	quantitative, cross-sectional studies (two cross-lagged samples pre- and during COVID) peer reviewed	change in workload, job satisfaction and occupational stress levels among Polish midwives working before and during the COVID-19 pandemic	Poland	n = 225 midwives	During the pandemic, midwives experienced higher levels of stress, with workload and personal COVID-19 infection being significant predictors. Job satisfaction mitigated stress, but decreased as workload increased. Working during the pandemic was an important predictor of stress
Jones et al., 2022 [8]	Midwives'and maternity support workers'perceptions of the impact of the first year of the COVID-19 pandemic on respectful maternity care in a diverse region of the UK: a qualitative study	qualitative study (structured individual interviews) peer reviewed	Impact of COVID-19 on maternity services and factors influencing midwives'and allied health professionals'perceptions of respectful maternity care	UK	n = 11 Midwives and maternity support workers	COVID-19 led to communication challenges, staffing pressures, reduced social support, and family separation in maternity care. PPE hindered communication, while virtual care raised concerns about privacy and identifying safety issues
Kawamura et al., 2021 [66]	Mentality of pregnant women and obstetric healthcare workers about prenatal SARS-CoV-2 testing: A regional survey over the first wave of the COVID-19 pandemic in Japan	quantitative, cross-sectional study peer reviewed	attitudes toward prenatal SARS-CoV-2 screening among obstetric healthcare workers	Japan	n = 287 midwives/nurses and n = 57 obstetricians at delivery facilities	Most midwives and nurses found prenatal PCR screening helpful in reducing anxiety and preventing nosocomial infections. However, obstetricians expressed concern about false-positive results leading to unnecessary medical intervention. Distrust of the accuracy of PCR testing was particularly high among obstetricians at COVID-19 receiving facilities
Kaya et al., 2022 [67]	Hesitancy towards a COVID-19 vaccine among midwives in Turkey during the COVID-19 pandemic: A cross-sectional web-based survey	quantitative, cross-sectional study peer reviewed	acceptance, hesitancy and barriers to COVID-19 vaccines among midwives	Turkey	n = 806 midwives	A considerable number of midwives have been exposed to COVID-19, with some experiencing infections and even deaths among family members. Respondents expressed doubts about the effectiveness, safety and clinical evidence of COVID-19 vaccines. Addressing vaccine hesitancy requires targeted education and awareness campaigns to build trust and promote vaccine acceptance among healthcare workers
Kissler et al., 2024 [68]	Perinatal Telehealth: Meeting Patients Where They Are	qualitative study (structured individual interviews) peer reviewed	perinatal providers'telehealth experiences during and after the COVID-19 pandemic	USA	n = 17 midwives and nurses (perinatal outpatient setting)	Healthcare providers welcomed telehealth despite concerns about accessibility and safety. The benefits outweighed the drawbacks, despite connectivity issues. Midwives expressed satisfaction with telehealth, which promoted equitable care, but also raised safety concerns. Key concerns included access, psychological safety, and rapport building
Klamroth-Marganska et al., 2021 [69]	Does therapy always need touch? A cross-sectional study among Switzerland-based occupational therapists and midwives regarding their experience with health care at a distance during the COVID-19 pandemic in spring 2020	quantitative, cross-sectional study peer reviewed	midwives'experiences in providing health care at a distance during the COVID-19 pandemic lockdown	Switzerland	n = 501 midwives	Midwives expressed mixed feelings about providing health care at a distance, with concerns about the limitations of providing comprehensive care due to lack of physical presence. Legal issues, reimbursement challenges, and reduced effectiveness were major hurdles. However, maintaining client relationships during the pandemic was seen as a significant benefit
Kornelsen et al., 2022 [70]	Care providers'experiences with and attitudes towards virtual antenatal care: Findings from a qualitative study in British Columbia	qualitative study (open-ended individual interviews and focus groups) peer reviewed	maternity care providers'experiences and attitudes towards virtual antenatal care in rural communities	Canada	n = 82 primary maternity care providers and urban and rural specialists	Participants highlighted the benefits of tripartite consultation in virtual care, involving the family physician or midwife in addition to the patient and a specialist, but noted increased workload and connectivity issues. Concerns included lack of physical exams and relationship challenges. Virtual care improved access but raised concerns about quality and workload. The institutionalization of fee codes for reimbursement of virtual care was recommended by the participants
Kucukturkmen et al., 2022 [71]	A qualitative study of Turkish midwives'experience of providing care to pregnant women infected with COVID-19	qualitative study (structured individual interviews) peer reviewed	experiences of midwives caring for COVID-positive pregnant women during labor and delivery	Turkey	n = 15 midwives	Turkish midwives faced challenges during COVID-19, emphasizing self-protection, isolation measures, and emotional distress. Variations in mother-baby contact and breastfeeding practices were observed. Professionalization and adaptation to new norms were noted. Midwives emphasized the need for evidence-based guidelines and clear admission protocols
Liebergall-Wischnitzer et al., 2023 [72]	A Correlational Study Of Midwives'Self-Compassion, Psychosocial Health, and Well-Being During the First Wave of COVID-19. What Have We Learned?	quantitative, cross-sectional study peer reviewed	midwives’ self-compassion, psychosocial health, and well-being, and their interrelationship during the initial COVID-19 wave	Israel	n = 144 midwives	Midwives showed high levels of burnout, with some considering resignation. Nevertheless, during the first wave of the pandemic, they demonstrated moderate to high levels of self-compassion and good psychosocial well-being, which correlated with years of experience and age. Self-compassion appeared to be central to maintaining mental health under stressful conditions
Lowe et al., 2020 [73]	Preparing maternity for COVID-19: A translational simulation approach	Description of an Intervention	Implementation of a translational simulation to improve maternity services preparedness for the COVID-19 pandemic by addressing emerging clinical priorities	Australia	Maternity simulations involving over 250 staff participants	The translational simulation effectively identified and addressed system changes for maternity care during COVID-19. Lessons learned included PPE challenges, communication difficulties, and emergency management adjustments. Simulation facilitated rapid solution development and team preparedness
Marin-Cos et al., 2022 [74]	Maternal Vaccination Greatly Depends on Your Trust in the Healthcare System": A Qualitative Study on the Acceptability of Maternal Vaccines among Pregnant Women and Healthcare Workers in Barcelona, Spain	qualitative study (structured individual interviews) peer reviewed	healthcare workers’ perceptions of maternal vaccines and motivational factors influencing vaccine decision-making	Spain	n = 14 healthcare workers in clinical setting (midwives, obstetricians and gynecologists)	Healthcare workers were reluctant to use COVID-19 vaccine in pregnancy due to uncertainties. Changing protocols led to uncertainty. Trustworthy guidelines are critical for healthcare workers to build positive relationships and improve vaccine uptake
Matthews et al., 2024 [75]	Midwifery workforce challenges in Victoria, Australia. A cross-sectional study of maternity managers	quantitative, cross-sectional study peer reviewed	The study examines the state of midwifery in Australia, including staffing, challenges and the impact of the COVID-19 pandemic, to identify gaps and assess the shortage of full-time midwives	Australia	n = 68 Midwifery managers	The findings revealed widespread recognition of inadequate midwifery staffing levels, exacerbated by the COVID-19 pandemic. Staff retention was influenced by concerns about job security, decisions to retire during COVID, and fluctuations in staffing due to lockdown measures. Regional and rural areas faced significant challenges in recruiting experienced midwives for provision of maternity care during the COVID-19 pandemic
McGrory et al., 2022 [76]	Self-Reported Experiences of Midwives Working in the UK across Three Phases during COVID-19: A Cross-Sectional Study	qualitative analysis of open-ended questions (cross-sectional study) peer reviewed	UK midwives'self-reported experiences of working during the COVID-19 pandemic	UK	n = 381 midwives	The study found that midwives experienced significant stress and a reduced quality of working life during the pandemic. This was primarily due to staffing shortages, partner restrictions, and service changes. These challenges were compounded by inadequate availability of personal protective equipment and constant changes in guidelines, which had a negative impact on midwives'mental health
McInally et al., 2022 [77]	COVID-19: obstetric sonographers'working experiences during the pandemic	qualitative study (structured individual interviews) peer reviewed	exploring obstetric sonographers'practices and experiences during COVID-19, focusing on their physiological and psychological well-being	UK	n = 6 obstetric sonographers	Participants experienced negative psychological responses due to COVID-19, such as fear of transmission, challenges with PPE, and difficulties with social distancing. Visitor restrictions had mixed impacts. Confusion arose due to PPE issues and changing guidelines. Participants emphasized the need for support, consistent guidelines, and improved working practices
Meloncelli et al., 2022 [78]	Clinicians'perspectives on gestational diabetes screening during the global COVID-19 pandemic in Australia	qualitative study (structured individual interviews) peer reviewed	perceptions and application of COVID-19-related changes to GDM screening and diagnostic recommendations, focusing on the appropriateness of the new guidelines from the provider's perspective	Australia	n = 17 healthcare professionals responsible for diagnosing or caring for women with GDM (midwives, nurses, endocrinologists, general practitioners, obstetricians, diabetes educators, dietitians)	Clinicians welcomed the initial changes in COVID-related GDM screening, but they questioned the evidence and consistency of implementation as the pandemic threats decreased
Melov et al., 2022 [79]	Exploring the COVID-19 pandemic experience of maternity clinicians in a high migrant population and low COVID-19 prevalence country: A qualitative study	qualitative study (structured individual interviews) peer reviewed	maternity clinicians'experiences serving a highly immigrant population during the COVID-19 pandemic, including patient perceptions, service delivery, and personal experiences amidst movement restrictions and border closures	Australia	n = 14 maternity care clinicians (midwives, medical doctors)	COVID-19 travel restrictions caused loss of family support for migrants. Clinicians shifted focus to staff safety, impacting patient-centered care. Cultural nuances affect care needs. Anxiety increased due to pandemic challenges
Memmott et al., 2022 [80]	'Forgotten as first line providers': The experiences of midwives during the COVID-19 pandemic in British Columbia, Canada	qualitative study (structured focus groups and in-depth interviews) peer reviewed	frontline experiences of midwives during COVID-19 in British Columbia, Canada, with the aim of understanding the challenges faced and strategies to support the well-being and health of patients	Canada	n = 13 midwives	The findings highlight a significant lack of support and recognition for midwives during the pandemic, leading to burnout and staff shortages. Addressing these inequities is critical to sustaining midwifery and patient care
Menon et al., 2023 [81]	Maternity Care Clinicians'Experiences Promoting Infant Safe Sleep and Breastfeeding During the COVID-19 Pandemic	qualitative study (structured individual interviews) peer reviewed	clinicians'perceptions of promoting infant safe sleep and breastfeeding during COVID-19 in a national quality improvement intervention	USA	n = 29 clinicians in maternity care (Registered nurses, certified nurse-midwives, lactation consultants, medical doctors)	Clinicians were burdened by hospital policies, coordination, and capacity constraints. Isolation affected parental interactions during labor. After-care formats were reconsidered, and shared decision-making about infant safe sleep and breastfeeding was emphasized amid COVID-19 uncertainty
Moltrecht et al., 2022 [93]	Challenges and opportunities for perinatal health services in the COVID-19 pandemic: a qualitative study with perinatal healthcare professionals	qualitative study (structured individual interviews) peer reviewed	impact of COVID-19 on perinatal services and health professionals caring for young parents under the age of 26, focusing on challenges and strategies in the midst of the pandemic	UK	n = 17 perinatal healthcare professionals	Perinatal professionals identified increased needs and challenges due to COVID-19, including reduced access to support, increased workload, and difficulties with remote care. Blended approaches were recommended to effectively address these issues
Nazik et al., 2021 [83]	Determination of Attitude and Stress Levels of Midwives, Nurses and Physicians Working in Obstetrics and Gynecology Clinics Regarding COVID-19 Pandemic	quantitative, cross-sectional study peer reviewed	Attitudes and stress levels of gynecology and obstetrics healthcare workers towards COVID-19, focusing on compliance with protective measures	Turkey	n = 134 healthcare workers in obstetrics and gynecology/clinical setting (midwives, physicians and nurses)	Healthcare professionals showed moderate worries, fear of infection, and stress levels toward the COVID-19 pandemic. Factors influencing stress levels included education, gender, and fear of contracting or transmitting the virus
Power et al., 2022 [84]	"Stranger in a mask"midwives'experiences of providing perinatal bereavement care to parents during the COVID-19 pandemic in Ireland: A qualitative descriptive study	qualitative study (structured individual interviews) peer reviewed	midwives'experiences of providing perinatal bereavement care during COVID-19, focusing on barriers, facilitators of care, and unprecedented challenges for grieving families	Ireland	n = 11 midwives providing bereavement care	Midwives faced challenges in providing compassionate bereavement care during COVID-19, including disruptions due to infection control measures and restrictions on human contact. Various strategies were employed, but barriers such as visitor restrictions and PPE hindered the provision of care
Riggan et al., 2021 [85]	Impact of the COVID-19 Pandemic on Obstetricians/Gynecologists	mixed methods cross-sectional study (descriptive analysis and thematic analysis of free-text responses) peer reviewed	impact of the COVID-19 pandemic on obstetricians and gynecologists in provision of care, focusing on policy changes, safety protocols and working conditions	USA	n = 72 obstetricians and gynecologists	The COVID-19 pandemic exacerbated stress among obstetricians and gynecologists due to policy changes, PPE shortages, and fear of exposure. Workplace pressures led to anxiety, fatigue, and sleep disturbances. Many considered leaving the profession. Support from family, colleagues, and resiliency measures helped, but long-term support is required
Rijnders et al. 2021 [86]	Centering in times of the COVID-19 pandemic	Qualitative evaluation of a centering intervention through anecdotal reports	Implementation of center-based antenatal care during the COVID-19 pandemic and its impact on supporting midwives and mothers amid lockdown	Netherlands	unclear (n = 101 midwives participated in the online workshop of the intervention)	The introduction of online centering sessions during the pandemic facilitated interactive learning and peer support. Midwives found typical group dynamics achievable online, increasing accessibility for women
Salameh et al., 2023 [87]	Trauma-informed care for perinatal women during the COVID-19 pandemic: A survey of nurses and midwives in Turkey	quantitative, cross-sectional study peer reviewed	maternal concerns and anxieties during the COVID-19 pandemic and aspects of trauma-informed care from the perspective of midwives and nurses providing perinatal care	Turkey	n = 102 participants midwives, perinatal nurses and nurse or midwife educators/managers	Maternity care providers identified vaccine safety among the top maternal concerns during COVID-19. Perceived nurse/midwife competence in trauma-informed care correlated with implementation. Barriers to trauma informed care included time constraints, lack of training, and conflicting guidelines. Promoting telehealth can support family-centered care
Scharmanski et al., 2020 [88]	Aufsuchende Familienbegleitung in der COVID-19-Krise durch Gesundheitsfachkräfte der Frühen Hilfen [Outreach support for families in the COVID-19 crisis by early help health professionals]	qualitative analysis of free-text responses from a cross-sectional study peer reviewed	healthcare professionals'perceptions of increased risk of violence in the families they serve during the COVID-19 pandemic	Germany	n = 58 community midwives and community health nurses	Community midwives and nurses shifted to telephone consultations during COVID-19, reducing home visits. This created challenges in maintaining support and quality of care, and increased maternal anxiety. Participants feared an increase in domestic violence and escalating conflict. Privacy issues and technical concerns with digital care were raised
Schmiedhofer et al., 2022 [9]	Birthing under the condition of the COVID-19 pandemic in Germany: Interviews with mothers, partners, and obstetric health care workers	mixed methods cross-sectional study (descriptive analysis and structured individual interviews) peer reviewed	first-hand experience of the impact of the COVID-19 pandemic on mothers, their partners, and obstetric professionals regarding birth and obstetric care in a university hospital	Germany	n = 10 obstetric professionals (midwives, doctors, nurses)	Maternity staff struggled to maintain usual standards of care in the midst of COVID-19. Security measures disrupted bonding time and raised concerns. Structural changes, including mandatory masks and limited visits, added to the burden. Fear of infection persisted. Exclusion of partners during work and visitor bans created moral dilemmas
Schmitt et al., 2021 [5]	Effects of the COVID-19 pandemic on maternity staff in 2020-a scoping review	scoping review peer reviewed	effecs of the COVID-19 pandemic on maternity staff	OECD countries	54 English and German articles published between January 2020 and January 2021	This scoping review provides a comprehensive overview of COVID-19's impact on maternity staff, highlighting structural challenges like staffing shortages and organizational changes. It also reveals significant negative effects on mental health, including increased depression, anxiety, and stress levels among the personnel
Selçuk Tosun et al., 2021 [89]	Anxiety levels and solution-focused thinking skills of nurses and midwives working in primary care during the COVID-19 pandemic: A descriptive correlational study	quantitative, cross-sectional study peer reviewed	state-trait anxiety levels and solution-focused thinking of midwives during the COVID- 19 pandemic and determinants of state-trait anxiety	Turkey	n = 91 midwives	Participants showed moderate anxiety and solution-focused thinking. State anxiety was inversely correlated with solution-focused thinking. Chronic illness and COVID-19 follow-up increased anxiety. Flexible work shifts improved solution-focused thinking. Perceived inadequacy of COVID-19 preventive measures increased anxiety
Silverio et al., 2023 [90]	Reflective, pragmatic, and reactive decision-making by maternity service providers during the SARS-CoV-2 pandemic health system shock: a qualitative, grounded theory analysis	qualitative study (structured individual interviews) peer reviewed	staff experiences of adapting maternity care policies during COVID-19 and maternity care staff decision making amid pandemic service changes	UK	n = 18 maternity staff (midwives and obstetricians)	The decision-making of maternity care staff during pandemic-related service changes fell into three categories: Reflective, Pragmatic, and Reactive. Reflective decisions promoted innovative, high-quality care. Pragmatic decisions, although justified, led to disruptions, particularly in virtual care and continuity of care. Reactive decisions devalued care and highlighted the need for consistent leadership and staff support
Simonovich et al., 2023 [104]	Qualitative Study of the Experience of Caring for Women During Labor and Birth During the First Wave of the COVID-19 Pandemic	qualitative study (structured individual interviews) peer reviewed	experiences of labor and delivery nurses and certified nurse midwives who cared for women in labor and delivery during the first wave of the COVID-19 pandemic	USA	n = 19 (labor and delivery nurses and certified nurse midwives)	Participants expressed distress at the separation of COVID-19-positive mothers and newborns, highlighting emotional challenges and policy inconsistencies. Isolation of women in labor was reported, exacerbated by limited communication and disparities in access to care. Maternity care providers faced mental health stress due to pandemic-related uncertainties, highlighting the need for improved support
Skelton et al., 2023 a [91]	UK obstetric sonographers'experiences of the COVID-19 pandemic: Burnout, role satisfaction and impact on clinical practice	quantitative, cross-sectional study peer reviewed	pandemic experience of obstetrical sonographers and assessing the impact of burnout using the Oldenburg Burnout Inventory for exhaustion and disengagement and the Clinical Outcomes in Routine Evaluation 10 for psychological distress	UK	n = 89 obstetric sonographers	Most sonographers experienced burnout, which was associated with negative impact on practice and decreased role satisfaction. COVID-19 moderately affected scanning practice and parent experience. High burnout rates had implications for workforce and service delivery. Many sonographers considered leaving the profession due to working conditions
Skelton et al., 2022 [82]	The impact of the COVID-19 pandemic on clinical guidance and risk assessments, and the importance of effective leadership to support UK obstetric sonographers	quantitative, cross-sectional study peer reviewed	COVID-19's impact on clinical practice and obstetric ultrasound services among UK sonographers, focusing on guideline implementation, risk assessment and perceptions of support during the pandemic	UK	n = 138 obstetric sonographers	Participants surveyed on COVID-19 guidelines, risk assessment and support faced challenges in implementing the guidelines due to environmental constraints. They felt most supported by ultrasound colleagues, but least by senior management and professional organizations. The guidelines were perceived as neither helpful nor unhelpful, with different local interpretations affecting risk reduction strategies
Skelton et al., 2023 b [94]	It has been the most difficult time in my career": A qualitative exploration of UK obstetric sonographers'experiences during the COVID-19 pandemic	qualitative analysis of free-text responses from a cross-sectional study peer reviewed	lived experiences of UK obstetric sonographers performing pregnancy ultrasound scans during the pandemic	UK	n = 111 obstetric sonographers	The pandemic had a profound impact on obstetrical sonographers'role dissatisfaction, perceived lack of support, moral injury, and psychological distress. Participants felt excluded from decision making, experienced moral injury due to ineffective leadership, and faced challenges in maintaining continuity of care. Despite this, they remained committed to providing high-quality, parent-centered care
Song et al., 2022 [95]	"This has definitely opened the doors": Provider perceptions of patient experiences with telemedicine for contraception in Illinois	qualitative study (structured individual interviews) peer reviewed	provider perspectives on the impact of telemedicine on patient care in contraception provision, focusing on access, counseling, privacy, and provision of long-acting reversible contraception	USA	n = 40 obstetrics-gynecology and family medicine physicians, midwives, nurse practitioners, and support staff	Providers viewed telemedicine for contraception positively, citing improved access and counseling. However, disparities and privacy concerns remained. Virtual visits offered flexibility but raised barriers, requiring equitable access and privacy solutions for future implementation
Steward, 2023 [98]	Interprofessional team trust in maternity services: a service evaluation	qualitative study (structured individual interviews) peer reviewed	participants examined the impact of a virtual maternity multidisciplinary forum,"maternal medicine huddle,"during COVID-19 on trust and organizational culture in maternity care, aiming to address issues of transparency and interprofessional relationships	UK	n = 6 midwives and obstetricians	Multidisciplinary virtual maternity forums fostered trust, improved communication, and enhanced teamwork despite interprofessional and interorganizational tensions. Participants noted a positive impact on women's experience of care and recommended that such forums be integrated into maternity systems for staff development
Stulz et al., 2022 [96]	Midwives providing woman-centred care during the COVID-19 pandemic in Australia: A national qualitative study	qualitative study (structured individual interviews) peer reviewed	midwives'experiences of providing woman-centered care during the COVID-19 pandemic and to identify lessons learned from government and hospital restrictions	Australia	n = 26 midwives	Midwives faced challenges due to COVID-19, adapting care in the midst of evolving policy. Limited time and telemedicine hindered woman-centered care, affecting relationships and support for families. Midwives innovated to maintain quality care, but some continuity models were reduced, highlighting the importance of autonomy
Talmont & Vitale, 2022 [97]	Telehealth Readiness Assessment of Perinatal Nurses	quantitative cohort study peer reviewed	Assessment of perinatal nurses'attitudes toward telehealth based on surveys of attitudes toward technology and readiness for telehealth	USA	n = 52 perinatal nurses	Perinatal nurses had mixed feelings about the impact of telehealth on their practice. They perceived telehealth as potentially reducing disparities, although some expressed concerns such as misdiagnosis. COVID-19 accelerated the adoption of telehealth, which was seen as a time-saving tool and a critical link in healthcare delivery
Tan et al., 2024 [16]	Challenges of being a maternity service leader during the COVID-19 pandemic: a descriptive analysis of the journey	qualitative study with longitudinal design (structured individual interviews) peer reviewed	maternity service leaders'experiences during the COVID-19 pandemic, focusing on their perspectives and roles within health services	Australia	n = 11 maternity care leaders (midwives, medicals, nurses)	Maternity service managers were challenged during the pandemic to adapt rapidly to changing guidelines and policies. They played a critical role in decision making, service adaptation, information filtering, and staff support
Turner et al., 2022 [99]	A review of the disruption of breastfeeding supports in response to the COVID-19 pandemic in five Western countries and applications for clinical practice	review of pandemic-related changes in professional society guidelines on breastfeeding support peer reviewed	impact of the COVID-19 pandemic on breastfeeding support and professional guidelines	Australia, New Zealand, Canada, UK, USA	comparison of factual breastfeeding support during the COVID-19 pandemic in comparison to n = 19 professional society guidelines from USA, UK, Canada, Australia, New Zealand and WHO	Breastfeeding support during the pandemic varied by country. Mother-infant separation in the US correlated with lower breastfeeding rates. Virtual care increased, but posed challenges of equity and physical assessment. Guidelines varied from WHO recommendations, highlighting the need for standardized practices and improved access to virtual support
Umazume et al., 2021 [101]	The physical and mental burden on obstetricians and gynecologists during the COVID-19 pandemic: A September 2020 questionnaire study	quantitative, cross-sectional study peer reviewed	physical and psychological stress experienced by obstetricians and gynecologists in Japan during the COVID-19 pandemic, and identification of factors contributing to their psychological stress	Japan	n = 852 obstetricians and gynecologists	The majority of physicians experienced physical and psychological stress comparable to or greater than previous disasters. High stress was associated with regions with more infected patients and wearing heavy personal protective equipment, especially during routine deliveries, which increased the burden on obstetricians and gynecologists
Vanderlaan & Woeber, 2022 [100]	Early Perinatal Workforce Adaptations to the COVID-19 Pandemic	quantitative, longitudinal study peer reviewed	perinatal practice changes and workforce challenges during the first weeks of the COVID-19 pandemic to describe the health care system's response	USA	n = 181 providers of clinical perinatal maternity care (nurses, midwives, physicians)	Changes in practice during the COVID-19 pandemic varied among perinatal care providers. Shortages of staff and supplies were reported, with increased workloads observed in out-of-hospital settings. Systemic constraints exacerbated the challenges, highlighting the need for flexibility and equitable care
Wiltshire et al., 2021 [92]	To treat or not to treat: perceptions of the initial American Society for Reproductive Medicine COVID-19 recommendations among women's health providers	quantitative, cross-sectional study peer reviewed	health providers'perceptions of the initial American Society for Reproductive Medicine COVID-19 recommendations for infertility treatment and their attitudes toward pregnancy and fertility during the pandemic, focusing on the designation of infertility treatment as"elective"or"non-urgent"	USA	n = 127 healthcare providers of obstetrics and gynecology (physicians, mid-level providers and nurses)	Overall, respondents generally agreed with the initial recommendations of the American Society for Reproductive Medicine to suspend infertility treatment during COVID-19. Both younger respondents, aged 18–30, and physicians specializing in obstetrics or gynecology opposed refraining from planned conception during the pandemic, with the former supporting access to infertility care regardless of COVID-19 burden
Woeber et al., 2022 [102]	Midwifery Autonomy and Employment Changes During the Early COVID-19 Pandemic	quantitative, cross-sectional study peer reviewed	pandemic's impact on midwifery employment and autonomy from the perspective of lead midwives	USA	n = 727 lead midwives of midwifery practices	Midwifery practices experienced more losses than gains in employment and compensation during COVID-19. However, midwifery-owned and community-based practices showed a more robust midwifery workforce during the first 6 months of the COVID-19 pandemic. Advocates for robust perinatal care should consider various forms of midwifery autonomy to ensure workforce resilience
Zucco et al., 2021 [103]	Rapid Cycle Implementation and Retrospective Evaluation of a SARS-CoV-2 Checklist in Labor and Delivery	qualitative, retrospective implementation evaluation reporting Standards for Quality Improvement Reporting Excellence (SQUIRE) guidelines and the Template for Intervention Description and Replication (TIDieR) peer reviewed	identification of factors that impacted implementation of a new obstetric workflow checklist, specific for COVID-19 patients in the perioperative setting,	Israel	Labor and delivery unit (no fixed number of participants)	The implementation of processes and innovative features significantly facilitated the adaptation of healthcare practices during the COVID-19 pandemic. External pressures, communication barriers, and individual beliefs affected the implementation of a new obstetric workflow checklist. The findings underscore the importance of organizational culture and readiness for rapid change during crises

### Overview of identified themes

During the data extraction process, the included studies and their results were assigned to eight overarching themes, all reflecting the effects of the COVID-19 pandemic on maternity care. These themes are presented in Table 2:

**Table 2:** Overview of the identified themes

Theme	Description and content of the theme
I. Working conditions	addressing the impact of the increased workload and altered working environments for maternity staff
II. Mental health of maternity staff	highlighting the psychological impacts on those providing maternity care and exploring the ethical challenges faced by maternity staff
III. Mental health of patients	investigating the mental well-being of pregnant women and new mothers
IV. Vaccination against SARS-CoV-2	discussing the implications and uptake of COVID-19 vaccines
V. Organization of care	analyzing changes in the structure and delivery of maternity care; examining the implementation of guidelines and infection control measures during the pandemic and their effects on the provision of maternity care
VI. Quality of care during COVID-19	assessing the maintenance of care standards during the pandemic and evaluating the impact of limiting visitors on maternity wards
VII. Personal protective equipment (PPE)	considering the availability and use of PPE in maternity settings
VIII. Implementation of telehealth	reviewing the adoption and effectiveness of telehealth in maternity care

**Fig. 2:**
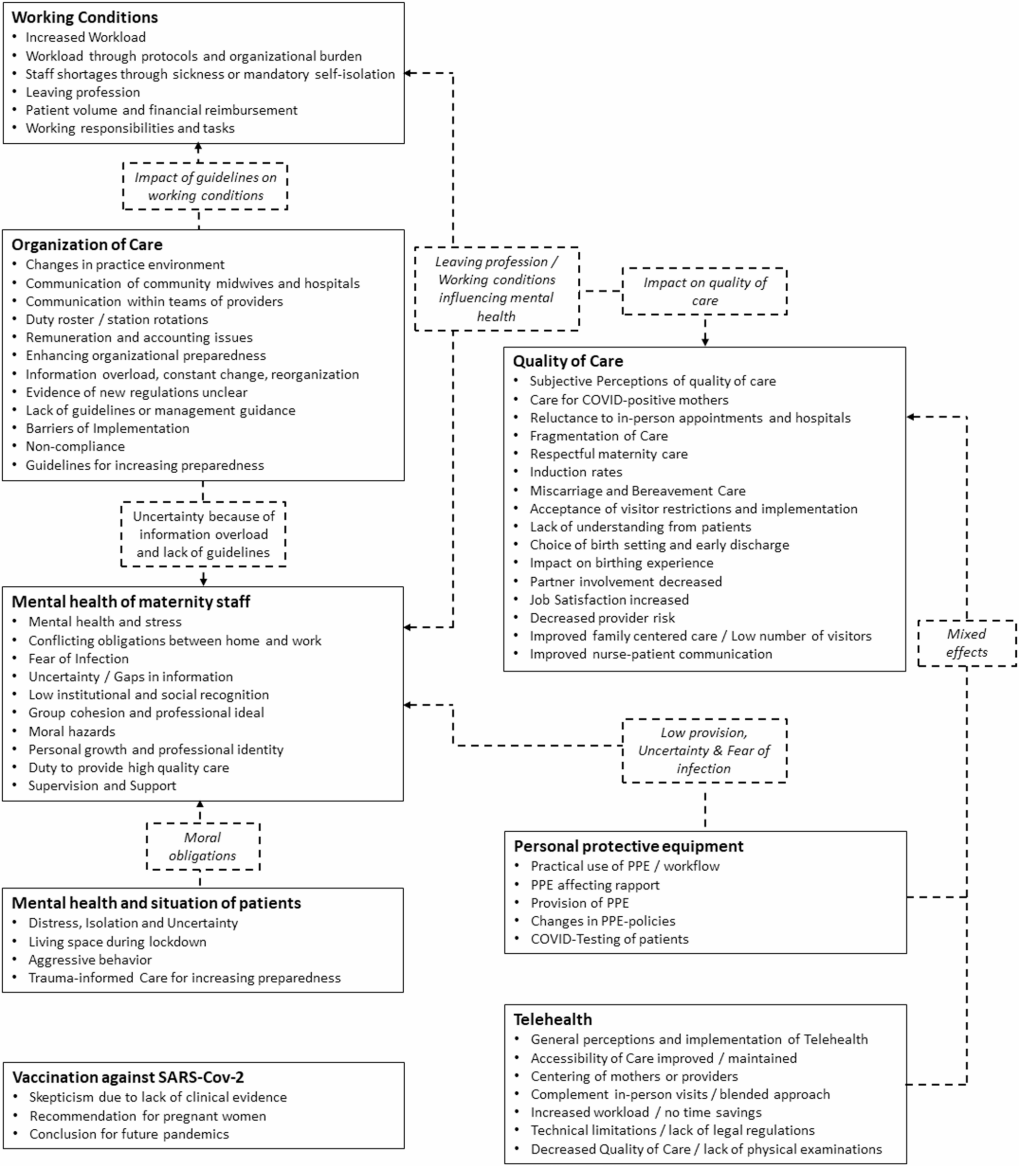
Overview of the emerging themes and their corresponding sub-themes

Each of these themes contained several subthemes, which are detailed in Fig. 2, together with the emerging overlaps and relationships between these themes. This thematic organization allowed for a comprehensive understanding of how the COVID-19 pandemic has affected different facets of maternity care, which will be discussed in detail below.

## Theme I. Workload and working conditions

The COVID-19 pandemic had a significant impact on the working conditions of maternity care providers, representing various sub-themes: increased workload and additional burden due to protocols and organizational changes, staff shortages due to illness or mandatory self-isolation, attrition from the profession, and lack of corresponding and financial reimbursement.

### Increased workload and additional burdens due to protocols

During the COVID-19 pandemic, maternity care providers experienced significant increases in workload. The implementation of new care practices, the need for longer and more frequent appointments due to heightened anxiety among pregnant and postpartum women, and the extensive use of PPE contributed massively to this increase [65, 79, 82, 83]. Many midwives reported working additional hours, often extending from the usual 8-h shifts to 12-h shifts [83]. The need to adapt cleaning regimes and manage frequent changes in regulations further exacerbated their workload [65, 79, 83]. The pandemic introduced numerous new protocols and organizational changes that added to the workload of maternity care providers. Midwives and other healthcare professionals had to rapidly implement and adapt to constantly changing guidelines related to PPE, protocols, and procedural changes [29, 30, 54]. These frequent updates created additional stress, as healthcare workers had to stay up-to-date with the latest requirements [82, 96]. The need to manage PPE and other bureaucratic tasks took time away from direct patient care, further increasing the organizational burden [81, 83]. Furthermore, healthcare providers had to take on additional responsibilities and tasks during the pandemic. This included performing duties typically handled by other hospital staff to minimize exposure risks [52]. Midwives and nurses also had to enforce infection control practices, which added to their workload and took time away from direct patient care [39]. The rapid changes in hospital policies and protocols required continuous adaptation and added stress [80, 85].

### Staff shortages through sickness or mandatory self-isolation

Staff shortages were a significant issue during the pandemic, exacerbated by sickness and mandatory self-isolation. These shortages resulted in some practices losing both full-time and part-time healthcare personnel, exacerbating the strain on the remaining staff and impacting the quality of care provided [104]. This led to increased stress and workload for the remaining staff, who had to cover for their absent colleagues[41].

### Leaving profession

The increased workload and stress led some healthcare providers to consider leaving the profession. Stress-related resignations were noted among midwives and nurses, with some opting for early retirement or career changes [8, 52, 71]. The additional responsibilities and emotional burden during the pandemic contributed to feelings of burnout and dissatisfaction [41, 75].

### Patient volume and financial reimbursement

The number of patients in maternity care settings remained relatively stable during the pandemic, unlike other healthcare areas such as elective surgeries [34]. However, the financial reimbursement for maternity care providers did not always match the increased workload and risks they faced. Some midwives reported doing unpaid work, such as providing free consultations before officially taking on clients [93]. There was also disappointment among midwives about the lack of financial support compared to other healthcare workers [80].

## Theme II: Mental health of maternity staff during the COVID-19 pandemic

This theme addressed the impact of working conditions during the COVID-19 pandemic on the mental health of maternity staff, focusing on stress, conflicting obligations between home and work, fear of infection, uncertainty due to gaps in information, and low institutional and social recognition. A number of studies also examined group cohesion and professional ideals during the pandemic. This theme further discusses the moral hazards faced by maternity providers during the COVID-19 pandemic. The pandemic posed significant ethical dilemmas, influenced personal and professional development, and underscored the commitment of maternity care providers to maintain high standards despite challenging conditions. Finally, various authors provide recommendations for future pandemics regarding the supervision and support of maternity staff.

### Mental health and stress

Maternity staff experienced high levels of stress, poor mental health, and burnout due to the challenging working conditions during the COVID-19 pandemic [10, 19, 33, 49, 101]. The uncertainty about the pandemic's progression and related professional and personal challenges exacerbated these stress levels [38, 43]. Many studies reported anxiety [5, 19, 33, 38, 40, 41, 49, 55, 56, 71, 82, 83, 85, 97], exhaustion [47, 52, 80, 93, 102, 103] and clinically relevant mental strain [46, 61, 72, 100] among maternity staff. Contributing factors included exposure to the virus, loss of colleagues, constant changes in work procedures, limited resources, and difficulties in caring for COVID-19 patients [10, 64, 82]. The tension between self-protection and fulfilling professional duties led to moral conflicts, significantly impacting staff [5, 34, 43].

### Conflicting obligations between home and work

Healthcare workers, especially in maternity care, faced challenges balancing private and family obligations with their work, adding personal stress during the pandemic [5, 19, 39, 40, 56, 58]. These challenges also affected their financial situations [10]. Hospital midwives reported to have shielded their families from workplace stress by downplaying their experiences [47, 82].

### Fear of Infection

Fear of COVID-19 infection significantly affected the mental health of healthcare staff [5, 37, 39–41, 47, 52, 56, 61, 64, 66, 80, 81, 84, 85, 92, 96, 97, 99]. They feared contracting the virus and transmitting it to family, colleagues, mothers, and newborns [5, 8–10, 19, 29, 33, 34, 39, 63, 65, 80]. Uncertainty about the efficacy of the protective equipment and risk of infection increased stress [52, 85, 96]. Despite personal risks, maternity staff maintained care for women and families while protecting themselves and their families from an infection [38, 49], heightening the levels of stress, anxiety, and depression [84, 92]. Isolation and measures to prevent family infection further strained the mental health of maternity staff [5, 80, 82, 83, 99], with loneliness being a significant factor [5, 7, 10, 19, 39, 46, 56, 82–85, 102].

### Uncertainty/gaps in information

Uncertainty and information gaps increased anxiety regarding infections, particularly due to constantly changing guidelines and inadequate information on virus transmission and the proper use of personal protective equipment [5, 8–10, 34, 39–41, 49, 52, 54, 57, 65, 71, 73, 81, 83, 84, 91, 98]. Frequent changes and unclear leadership responses intensified staff confusion and stress [7, 99].

### Low institutional and social recognition

Maternity staff felt underappreciated by their organizations, when they were initially forbidden to use masks at the beginning of the pandemic [19, 38, 39, 79, 102]. Poor communication from management on care changes increased feelings of abandonment [10, 63]. Authoritative leadership and lack of participation in decision-making heightened feelings of alienation [54]. Often, staff felt that their expertise was ignored in protocol development [80, 102] or their contributions were undervalued [76, 79].

### Group cohesion and professional ideal

Cohesion and commitment among colleagues were described as crucial in managing stress among maternity staff [10, 40, 53, 82, 91]. Peer support fostered a supportive atmosphere, which enabled resilience and adaptation through a positive work environment [10, 83]. Various studies pointed towards how a strong professional identity bolstered confidence and resilience among maternity staff [34, 54, 72].

### Moral hazards

The COVID-19 pandemic brought profound moral dilemmas and ethical challenges to maternity care providers. These dilemmas included balancing infection control measures with the provision of adequate maternity care [58]. Despite the fear of contracting COVID-19 and difficult working conditions, many midwives and obstetricians felt morally obligated to provide high-quality care [61, 63, 65, 80]. Especially restricting access for companions during childbirth was a significant issue, as it conflicted with professional standards and caused emotional distress for both staff and patients [9]. Reports highlighted the dehumanization of birth experiences due to constantly changing guidelines and the challenge of providing a safe and respectful environment for COVID-19 positive mothers [5]. Maternity staff perceived the separation of COVID-19 positive mothers from their newborns as an emotionally taxing and potentially harmful procedure to the mother–child bond [19, 92].

### Personal growth and professional identity

Some maternity care workers perceived the pandemic as a period of professional development, as they gained confidence in their abilities to manage uncertain situations [10, 71]. Many expressed pride in their professional roles, particularly those who could maintain high care standards or quickly adapt to new standards of care [91]. Initiatives like the AREU project in Italy, where midwives provided telephonic support to pregnant and breastfeeding women, were viewed as personally and professionally enriching [47]. The sense of solidarity among maternity care workers was strengthened by the shared challenges, fostering a supportive work environment [10, 30, 82].

### Duty to provide high-quality care

Despite the risk of infection, many maternity care workers prioritized their duty to provide adequate care, often at great personal cost [5, 10, 30, 38, 83, 102]. Their professional identities were deeply intertwined with their commitment to support mothers during childbirth, even under crisis conditions [19, 33, 85]. However, the inability to meet usual professional standards due to restrictions, such as the exclusion of partners during birth, posed significant emotional and ethical challenges [5, 9]. Some staff reported flexibly handling hygiene protocols to provide respectful care, even if it meant breaching infection control guidelines [9, 98].

### Supervision and support

Brown et al. [34] argued that future pandemic responses should include more resources, especially supervision for health staff, with continuous support to maintain well-being. This should encompass individual, interpersonal, and organizational interventions to reduce stress and build resilience. Providing psychological support and training is crucial for maintaining psychosocial health [43, 46]. Adequate resources, clear guidelines, and improved cooperation between professions can reduce workloads and improve staff situations [10, 41, 92, 93, 97].

## Theme III: Mental health of patients from the perspective of the providers

Various studies explored how maternity staff perceived the mental health and situations of patients during the COVID-19 pandemic, focusing on distress, isolation, uncertainty, living space during lockdown, and aggressive behavior. One particular Turkish study investigated the impact of the pandemic on the provision of trauma-informed care.

### Distress, isolation, and uncertainty

Maternity staff was afraid that young parents, especially new or expecting mothers, experienced heightened isolation and uncertainty during the pandemic, especially since face-to-face interactions, crucial before the pandemic, were replaced by online meetings [64]. Social isolation was exacerbated by lockdowns, limiting opportunities for young mothers to connect with peers about pregnancy and childcare [9]. Fear of infection led to increased appointment cancellations and early hospital discharges [8]. Many mothers were uncertain due to insufficient information about COVID-19, and a lack of personal PPE among healthcare workers further heightened this uncertainty [5]. Rapidly changing guidelines added to the confusion and frustration not only for healthcare providers but also for patients [41, 49]. From the perspective of obstetric staff, all these factors made it difficult to provide care during the pandemic [9, 64].

### Living space during lockdown

Midwives perceived home environments for many young mothers as overcrowded, creating significant challenges for clinical interactions and maintaining confidentiality during home visits. They feared that these cramped conditions compromised the quality of care and posed serious privacy issues [64]. The lockdown worsened these pre-existing issues, making it harder to find suitable spaces for necessary healthcare interactions.

### Aggressive behavior

Healthcare workers faced aggressive behavior from patients and their families, partly due to being perceived as carriers of the virus. This hostility included violent attacks and abusive behavior [97].

### Trauma-informed care

One study from Turkey examined the provision of trauma-informed care during the COVID-19 pandemic [87]. The authors highlighted the importance of implementing trauma-informed care practices to address maternal concerns and anxieties related to pregnancy and childbirth. Only a small percentage of Turkish healthcare providers used telehealth to explicitly address these concerns during the pandemic.

## Theme IV: Vaccination against SARS-CoV-2

This theme focused on skepticism about SARS-CoV-2 vaccination among maternity staff due to lack of clinical evidence, recommendations for pregnant women, and implications for future pandemics regarding vaccination.

## Skepticism due to lack of clinical evidence

In a survey conducted among Turkish midwives, a majority expressed skepticism towards receiving the COVID-19 vaccine themselves, with less than half considering vaccination [67]. The primary reasons for this skepticism were insufficient clinical studies and safety concerns about imported COVID-19 vaccines. Conversely, a study among French obstetricians reported a generally high willingness to get vaccinated against SARS-CoV-2 [42].

### Recommendation for pregnant women

Surveys conducted in Spain and France indicated that midwives and obstetricians generally supported vaccinating pregnant women against SARS-CoV-2, although some expressed various reservations [42, 67, 74, 87]. These reservations were due to a lack of data on adverse effects and vaccine efficacy, the need for additional information from professional associations, or greater fear of the vaccine than of SARS-CoV-2 itself [42, 87]. Consequently, despite different vaccination recommendations, COVID-19 vaccination was sometimes not recommended to pregnant women in the first and third trimesters. Frequent changes in guidelines during the pandemic led to uncertainties and doubts among maternity staff and pregnant women, significantly complicating vaccination recommendations and decisions [42].

## Implications for future pandemics

Midwives and nurses in maternity care perceived the close relationship between pregnant women and maternity staff, particularly midwives during prenatal care, as a significant factor in the acceptance and implementation of vaccination [42]. Support from professional associations and government institutions proved essential in encouraging pregnant women to get vaccinated [67, 87]. Improved education and evidence-based information can alleviate fears of adverse effects [42]. Therefore, it is important for healthcare professionals to provide women with accurate and reliable information about vaccines in case of future health crises.

## Theme V: Organization of care

A number of articles addressed the organization of care during the COVID-19 pandemic, focusing on changes in practice environments, communication among community midwives and hospitals, communication within teams and duty rosters. Studies also pointed towards remuneration issues and formulated recommendations for enhancing organizational preparedness. The implementation of guidelines during the COVID-19 pandemic significantly impacted the working conditions of maternity staff, primarily concerning information overload, constant change and reorganization of care, questionable evidence of new regulations, and a lack of management guidance. The included studies identified various barriers to the implementation of guidelines and explore reasons for non-compliance.

### Changes in practice environment

During the COVID-19 pandemic, infection control measures imposed significant organizational burdens and deviated from standard care. Strict access regulations and visitor restrictions posed challenges for both staff and patients [5, 9, 71]. Midwives encountered disorganized postpartum care, while obstetricians advocated for maintaining routine intrapartum care [29, 30]. Necessary organizational adaptations included ventilation improvements, restructuring waiting and examination rooms, redefining clinical pathways, creating isolated areas for infected women, and reallocating bed capacity [5, 7, 9, 48, 57, 79, 82, 96]. Implementing protective measures like social distancing was often difficult due to inadequate facilities and infrastructure [54, 57], exacerbated by the increasing number of COVID-19 positive pregnant women [41]. Furthermore, social distancing was not feasible for many obstetric examinations [97].

### Communication and cooperation between community midwives and hospitals

Numerous studies highlighted deficits in the communication and cooperation between hospital staff and freelanced midwives during the pandemic During the pandemic, the emphasis on clinical settings in maternity care led to tensions, particularly among midwives working outside clinical environments who experienced increased strain in their collaboration with clinically based physicians and midwives [53, 65]. While clinical midwives reported acceptable collaborations within their facilities, freelance midwives were often dissatisfied with hospital cooperation and struggled with referring women to other providers and hospitals [30, 50]. Several midwives experienced stigmatization from hospitals, related to misconceptions about midwifery work [93]. Further, midwives felt underrepresented in crisis teams, hindering interprofessional dialogue with senior physicians and infection specialists [29, 45]. Various studies argue that improved communication and cooperation between hospitals and independent midwives are crucial for ensuring care for pregnant women and newborns during crises [50, 53, 93].

### Communication within teams of providers

The communication within provider teams faced challanges during the pandemic as well. Tensions and mistrust between different professional groups hindered collaboration [10, 31, 39, 54, 63]. Innovative approaches like COVID-WhatsApp groups, online meetings, and workflow checklists were implemented for efficient communication [30, 79, 90]. Dedicated COVID-19 roles facilitated timely information sharing, supporting infection prevention guidelines [57]. Management played a key role in adapting care to governmental guidelines [7]. Clear and effective communication was vital for the support of the management, with staff feeling supported when supervisors were present and provided clear directives [41].

### Changes in duty roster/station rotations

Staff reported significant flexibility in duty rosters [30] or experienced voluntary or forced shift reductions [34] during the pandemic. Obstetric staff were often reassigned to COVID-19 care, causing shortages in regular maternity care [79]. This included reassignment to other departments, conducting COVID-19 screenings in unfamiliar teams, and merging departments [6, 41, 52]. However, these reallocations of staff between departments significantly increased workloads [64]. Early retirements, reassignment due to pre-existing conditions, and mandatory quarantines exacerbated staffing shortages [85]. These challenges were compounded by pre-pandemic weaknesses in maternity departments, such as staffing shortages and frequent personnel changes and hospitals had to organize flexible duty rosters and form new teams under tight deadlines [9]. In Germany, volunteers were matched with hospitals through an internet platform, retired staff were reactivated, and students were employed for paid internships to address staffing shortages [5]. Similarly, extended placements and deployment of students in the NHS workforce were implemented in order to compensate for the increased workload during the COVID-19 pandemic [36].

### Remuneration and accounting issues

The financial and accounting challenges arising from the pandemic included income losses and structural difficulties in the provision of healthcare. Studies describe the financial impact on maternity care, including reduced check-up visits and hospital staff shifts, leading to financial strain [34, 46]. Midwives often operate within a competitive financial environment that does not prioritize interpersonal care of mothers, exacerbated by inadequate government investment during the pandemic [10, 38]. A French study contrasted the financial impact on independent and employed midwives [50]. While independent midwives largely continued to work during the closure, employed midwives temporarily stopped work to reorganize services, with independent midwives being more flexible in responding to challenges.

### Information overload, constant change, and reorganization

A significant challenge faced by maternity care staff was the constant change and reorganization of care driven by an overwhelming amount of information. The guidelines in maternity care were marked by constant updates and restructuring, requiring rapid adaptations to new clinical processes and structures [9, 57, 58, 64, 83]. This led to confusion and distrust among staff, due to the frequent changes in protocols and guidelines [97]. Communication of accurate information was also difficult due to these ongoing changes [8]. The inconsistencies and frequent updates of guidelines induced confusion and uncertainty among maternity staff [5, 10, 31, 78, 81, 91]. This was particularly problematic when contradictory guidelines existed between hospitals, outpatient care, and different specialties, particularly concerning postpartum care [30, 57, 71, 79]. These discrepancies raised concerns about the quality of care among maternity staff [91]. The constant reorganization led to increased burden as routine tasks became more complex, requiring additional time and focus [38, 40, 63, 82]. The robustness of new guidelines was often perceived unclear by maternity staff, as many were not evidence-based and provided limited information [39, 71, 82]. Clinicians expressed concerns about the lack of transparency and reliability of new data, prompting some to seek other official sources with stronger evidence bases [78]. Uncertainty also existed regarding the use of published guidelines to support departmental protocols, leading some departments to develop their own guidelines [48]. Fumagalli et al. [82] recommend preparing unified and comprehensive guidelines for future health crises, particularly in the event of drastic reorganization of maternity services. These guidelines should account for the specific needs of maternity care, especially woman-centered and respectful care. Additionally, midwives should receive resources and training for implementing telehealth services to ensure the inclusion of birth partners when physical presence is not possible.

### Lack of guidelines or management guidance

Maternity care staff reported a lack of clear guidance from their supervisors, leading to uncertainty [29, 31]. This lack of support and effective management from hospital administrations or superiors resulted in stress and frustration among staff [48, 85, 102]. Midwives, overwhelmed by various guidelines, particularly those perceived as non-evidence-based, often relied on pre-pandemic existing and evidence-based guidelines to ensure safe, patient-centered care [98]. The rapidly changing situation required swift decisions from leadership, but the flood of information and lack of clear guidelines complicated the adaptation to new measures [7]. Additionally, discrepancies between national guidelines and WHO recommendations led to further uncertainty in practice [89].

### Barriers to implementation

Implementing guidelines in maternity care faced several barriers, including contradictory information and practices, delays in implementing infection prevention and control measures, and difficulties in sourcing and using personal protective equipment [57, 79]. Clinicians struggled with the changing recommendations due to confusion over their meaning, ensuring consistency, and practical organizational issues [78]. Midwives found it challenging to keep up with constantly updated guidelines, preventing uniform implementation in practice [65]. Other barriers included constraints in the clinical work environment, such as space limitations, time pressures, and ventilation issues, as well as difficulties in filtering and translating information for staff and patients [7, 48]. General guidelines often did not cater to the specific needs of maternity care, where unavoidable contact with mothers and newborns and the inclusion of companions are essential, leading to unclear and challenging implementation [48, 57, 98].

### Non-Compliance

Non-compliance with guidelines by healthcare staff occurred in various situations and settings. For example, some obstetric practices deviated from national guidelines by allowing partners to accompany pregnant women despite recommendations that stated the opposite [30]. Factors contributing to non-compliance included uncertainty and poor communication [52], non-adherence to social distancing and PPE regulations [57], and concerns about evidence-based practices and the perceived threat of COVID-19 [78]. Moral considerations [80] and the desire for woman-centered care [98] also led midwives to bypass rules and push boundaries to ensure high-quality care despite pandemic fears. The fear of infection and the stress of rapidly changing guidelines further influenced adherence [90]. Patients and visitors were also found to deviate from infection measurements, frustrating and challenging healthcare workers [9, 63, 85]. Some pregnant women and their partners ignored COVID-19 safety measures, attending appointments with symptoms despite explicit prohibitions [65].

### Enhancing organizational preparedness

The pandemic revealed significant gaps in the preparedness and adaptability of health systems, particularly in maternity care. Pre-existing structural problems, chronic understaffing, and bureaucratic hurdles hindered efficient work and led to feelings of marginalization and lack of appreciation among professionals [38, 54]. This"endemic precariousness"resulted in a fragile and overstretched infrastructure, exacerbated by the shock of the pandemic [54]. Constant policy changes increased the pressure on staff. Despite occasional opportunities for innovation and transformation, these were often implemented through a top-down approach that was not always well received [54]. Structural improvements are needed to increase the resilience and preparedness of the health care system for future crises, including an infrastructure that allows for social distancing [57], the use of translational simulations to identify safety and performance issues in crises [73], and the provision of timely emergency training [5].

## Theme VI: Quality of care during COVID-19

This theme addresses subjective perceptions of maternity staff regarding the impact of the COVID-19 pandemic on quality of care, care for COVID-positive mothers, patients’ reluctance to in-person appointments, fragmentation of care, respectful maternity care, induction rates, and the provision of miscarriage and bereavement. Perceived disadvantages of visitor restrictions included a lack of understanding from patients, changes in birth setting choices and early discharge, a negative impact on the birthing experience, and decreased partner involvement. Frequently cited advantages from the perspective of maternity staff included increased job satisfaction, decreased provider risk, improved woman-centered care, and improved staff-patient communication.

### Subjective perceptions of quality of care

Various studies reveal the COVID-19 pandemic's impact on maternity care quality, highlighting visitor restrictions, COVID testing, personal protective equipment, continuity of care, service restructuring, and changes in patient volume [5, 34, 50, 65]. Despite these challenges, many staff members expressed satisfaction with the general quality of care provided [6, 33, 98] and saw the pandemic as an opportunity for innovative solutions like online meetings and telework [29, 91]. However, limitations in care due to contact restrictions and hygiene protocols were noted [34, 65, 79]. Reduced home visits, fewer check-ups, and inequities in access to care were reported, particularly for disadvantaged groups [34, 41, 59, 60, 80, 91, 92]. Increased workload due to longer hours and additional responsibilities also significantly impacted quality of care [8, 63, 71]. Elective procedures like in vitro fertilization were initially deprioritized [94].

### Care for COVID-positive mothers

Pregnant women were isolated upon admission to await COVID-19 test results, causing spatial distancing by staff [92]. There were concerns that these changes hindered essential support for new parents, exacerbating existing inequalities [64]. Treatment delays occurred as COVID-positive women were often seen at the end of shifts [8]. Despite hospital policies, some staff prioritized supporting laboring women over social distancing [92].

### Reluctance to in-person appointments and hospital-visits

Maternity staff observed that during the pandemic, patients were reluctant to attend face-to-face appointments and hospital visits for fear of infection [6, 10, 34, 65, 79]. Women delayed hospital visits, and missed check-ups and vaccinations were common [10, 65]. Preference for home births or birthing centers increased [6]. Early discharge requests were common, often due to restrictions on hospital visitors [30, 96].

### Fragmentation of care

The pandemic significantly fragmented care, disrupting usual clinical practices and continuity, increasing the burden on medical staff [6, 52, 54, 93, 102, 103]. Additional tasks diverted staff from patient care, straining patient relationships and negatively impacting quality of maternity care [52, 63, 91, 98].

### Respectful maternity care

The shift from woman-centered to staff safety-focused care reduced patient autonomy and increased paternalistic practices [58]. Limited presence of patients’ support persons reduced personal contact negatively impacted maternity care [8, 19, 33, 96, 98]. Challenges in respectful communication and the separation of COVID-positive mothers from their newborns were significant issues [8, 30, 92, 99], leading to reduced breastfeeding initiation and duration [71, 81, 89].

### Induction rates

Hospital reports on induction rates varied, with some noting decreased planned inductions, while others saw increased demand from healthy women seeking immediate labor initiation [34]. Protocol changes and shorter postpartum stays for COVID-positive women influenced induction and Cesarean-section rates [5, 6].

### Miscarriage and bereavement care

Social distancing significantly impacted miscarriage and bereavement care, restricting access to support and disrupting traditional mourning practices [32, 76]. Infection control measures severely limited individual bereavement support [84].

### Acceptance of visitor restrictions and implementation

Visitor restrictions during the COVID-19 pandemic were seen as necessary by maternity staff but were also evaluated as a harsh measure [35]. Most preferred a low number of visitors, averaging 0.9 per patient [37], despite the potential emotional burden on mothers and the possibility of women choosing to give birth elsewhere [45]. Experiences varied globally, with differing policies on birth support and prenatal visits [5, 57]. Some providers advocated for flexible handling of visitor policies [44]. Despite challenges, staff reported positive aspects, such as fostering closer bonds with patients, although it also increased pressure on staff to provide emotional support [5, 63].

### Lack of understanding from patients

Initially, patients and partners struggled to understand and accept visitor restrictions, requiring significant effort from staff to enforce and communicate these rules [9].

### Choice of birth setting and early discharge

Visitor restrictions led to an increase in home births and use of birth centers, as well as early hospital discharges, which many healthcare providers deemed medically critical due to reduced time for postnatal care [9, 35, 37, 45].

### Impact on birthing experience

Restrictions negatively affected birthing experiences, bonding, and early parenthood. COVID-positive mothers often had to give birth alone, without partner support, increasing emotional distress [6, 96]. While some families appreciated the isolation for bonding, many found it burdensome. Partners felt excluded from significant life events [49, 65]. Maternity staff reported that the absence of partners during key moments and fewer ultrasound visits were significant issues for patients, impacting their mental health and emotional well-being [8, 9, 41, 52, 63, 98]. Within an Australian study [49], maternity staff reported that travel restrictions due to COVID-19 severely limited family support, particularly impacting migrant women who relied on overseas relatives. This not only affected the mental health of these women but also increased the workload for medical personnel, as they had to provide additional assistance and support that would typically be offered by the family.

### Decreased partner involvement

The pandemic led to a significant decline in partner involvement and family support during pregnancy and birth. Midwives and obstetricians complained about a reduced partner participation in prenatal care and ultrasound visits, largely due to restrictions on accompanying persons to minimize infection risks [30, 32, 34, 35, 44, 45, 79, 81, 82, 96].

### Job Satisfaction increased

Despite the challenges, visitor restrictions sometimes increased job satisfaction among maternity care staff, allowing for more focused care and patient education in a quieter environment. Many staff members favored maintaining these restrictions post-pandemic due to their perceived benefits [35, 44, 63]. Furthermore, visitor restrictions reduced COVID-19 infection risks for healthcare providers [37]. From the viewpoint of maternity staff, fewer visitors improved the quality of care by reducing distractions during emergencies and promoting better mother-infant bonding and breastfeeding practices [8–10, 34, 35, 44, 58, 63, 97]. A quieter environment also encouraged mothers to make autonomous decisions about their birth, independent of relatives [8, 9, 30, 44, 82, 98].

### Improved nurse-patient communication

Visitor restrictions allowed for better staff-patient communication, providing more time for uninterrupted discussion and effective patient education, improving patient rapport and quality of care [35, 52, 63, 79, 81].

## Theme VII: Personal protective equipment

Numerous studies have examined the use PPE during the COVID-19 pandemic in maternity care. Their scope included the practical use and workflow implications of PPE, the impact of PPE on patient rapport, the provision of PPE, changes in PPE policies, and COVID-19 testing of patients. The findings revealed significant challenges and adaptations to ensure safety and maintain quality of care.

### Practical use of PPE/workflow

The extended use of PPE imposed considerable physical strain on maternity care staff.

[19, 34, 64, 71, 82, 101]. Donning and doffing PPE were time-consuming and added to the workload, reducing time available for direct patient care [41, 52, 85, 96]. This led to delays, particularly concerning in emergency situations, where quick response was critical [5, 8].

### PPE affecting Rapport

The use of PPE, especially face masks, hindered non-verbal communication, essential for building trust between midwives and mothers [5, 6, 8, 9, 19, 29, 34, 52, 58, 63, 79, 80, 82, 84, 85, 91, 93, 96, 97, 103]. Midwives reported difficulties in reading facial expressions, leading to a general decrease in patient rapport [5, 58, 79, 84, 96]. Staff perceived PPE as an emotional barrier that impacted relationships with patients [8, 34, 82, 84, 91, 97]. The restriction of mimicry and body language by PPE made establishing connections and building relationships particularly challenging [5, 29, 34, 52, 80, 91, 93].

### Provision of PPE

Providing sufficient PPE for maternity care workers was a major challenge during the pandemic [5, 7, 30, 34, 38, 39, 41, 45, 52, 57, 63, 65, 79, 80, 83, 85, 93, 96, 99]. Shortages led to stress and uncertainty among staff, who often had to reuse PPE or source it themselves [5, 30, 39, 52, 65, 80, 83, 93]. Particularly, community maternity practices struggled to obtain PPE promptly [5, 45, 57, 79, 80, 93, 96]. These shortages negatively affected staff safety and well-being, increasing exposure risks [5, 45, 52, 65, 79, 80, 83, 85, 93, 96]. Experiences with PPE shortages led to calls for better preparedness and PPE provision, especially for freelanced midwives, to ensure their safety [7, 30, 34, 45, 65, 93, 96].

### Changes in PPE policies

During the COVID-19 pandemic, PPE policies underwent rapid changes as recommendations on the type, amount, and timing of protective gear frequently shifted due to resource fluctuations and new insights into risks of transmission [34]. These constant revisions were often viewed negatively by maternity care staff, adding to their burden [54]. For instance, in the UK, NHS staff were initially discouraged from wearing masks to avoid alarming patients at the beginning of the pandemic in 2020 [19]. Proper PPE use was a significant factor in the workload of maternity care providers. Uncertainties in PPE use, particularly at the onset of the pandemic, were compounded by a lack of studies on PPE's protective efficacy [96]. Healthcare workers who subjectively felt protected by PPE tended to experience less psychological stress [5, 40, 56, 96]. Regular COVID-19 testing of healthcare staff also contributed to greater confidence in PPE effectiveness [5, 82].

### COVID-testing of patients

The provision of COVID-19 testing and patient education about the tests posed additional challenges in maternity care during the pandemic. Maternity staff reported that mothers and midwives had inadequate access to COVID-19 tests and limited knowledge about available test resources and patient education [45]. Maternity care workers were aware of the limitations of prenatal testing and the risks involved, expressing concerns about excessive medical interventions due to false-positive results [66]. Maternity staff had to deal with many mothers who refused COVID-19 testing for a variety of reasons, including fear of separation from their newborns and distrust of the government [45]. While many professionals acknowledged the benefits of prenatal testing for reducing anxiety among staff and patients, not all felt secure. One Japanese study [66] highlighted differences in the acceptance of prenatal testing, with staff at hospitals specializing in COVID-positive mothers being less supportive of prenatal tests.

## Theme VIII: Implementation of telehealth

The final theme focused on the implementation and impact of telehealth during the COVID-19 pandemic. Maternity staff perceived advantages through telehealth because it improved accessibility, allowed for the centering of mothers and providers, and was generally seen as useful in its complementary role with in-person visits. However, disadvantages of telehealth included increased workload, technical limitations, and decreased quality of care.

### General perceptions and implementation of telehealth

The COVID-19 pandemic significantly accelerated the acceptance and implementation of telehealth, leading to a lasting transformation in maternity care [6, 10, 30, 41, 51, 64, 68, 79]. For instance, in Germany, digital midwifery services were permanently integrated into standard care by the Digital Healthcare and Nursing Care Modernization Act (DVPMG), a health policy and legislative decision [59, 60]. Telemedicine became a crucial measure for infection prevention and maintaining care continuity during the pandemic [5, 51, 59, 60, 69, 88]. While beneficial for low-risk births, telemedicine was perceived as less suitable for high-risk pregnancies or those with psychosocial or domestic issues [58, 69]. Skepticism remained among midwives and obstetricians, citing technical limitations, lack of quality standards, insufficient integration with in-person care, and perceived lower quality of communication compared to face-to-face interactions [10, 29, 58, 60, 69, 70, 85, 93]. However, telehealth improved access and efficiency, especially in rural areas and breastfeeding support [89]. Younger midwives in particular were in favor of implementing digital care after the pandemic [51, 59, 60, 69, 77].

### Accessibility of care was improved or maintained

Telehealth maintained or significantly improved access to gynecological and obstetric care by reducing barriers such as spatial distance, transportation issues, and childcare responsibilities [8, 70, 93]. Studies highlighted time savings and increased flexibility for providers [30, 60, 62, 65, 68–70, 77]. Telehealth facilitated scheduling of appointments and reduced travel time for patient contacts, particularly in rural or hard-to-reach areas [58, 62]. Telehealth also offered valuable insights into patients'home environments, beneficial for postpartum care [68].

### Centering of mothers or providers

During the pandemic, virtual meetings with pregnant women and those who have given birth, were organized in various countries [5]. A Dutch project successfully implemented virtual group sessions and additional consultations on COVID-related topics, promoting mutual support and network strengthening among mothers [86]. Steward et al. found that multidisciplinary virtual birth forums improved interprofessional teamwork and cross-organizational communication during the pandemic [53].

### Telehealth as a supplement to personal visits

Midwives viewed telehealth as a time-efficient complement to in-person care but emphasized that the option for in-person consultations was essential for the success of digital care [8, 38, 79]. A combination of telephone and in-person patient contacts within a blended approach was seen as useful [60, 62, 64, 70].

### Increased workload/no time savings

Despite some studies indicating time savings through telehealth, others reported increased workloads. Providers found telephonic consultations unexpectedly time-consuming due to the need for detailed explanations usually conveyed via computer or brochures [65]. The reimbursement and billing process for telehealth was globally seen as cumbersome and sometimes unfair for community midwives [10, 59, 60, 69, 70]. The rise in community midwifery work led to additional unpaid work without adequate resources [5, 10]. Virtual group meetings also required more time for administrative tasks [70]. Transitioning to hybrid care models necessitated comprehensive reorganization and coordination with other health professions [98].

### Technical Limitations and lack of legal regulations

Barriers for the use of telehealth included technical challenges and lack of support through specialized training and guidelines [60, 62, 70]. Technical issues like slow internet and unreliable connections caused frustration, especially in rural areas with limited broadband access [62, 68, 70, 91]. Providers faced data protection uncertainties and billing issues, leading to extra workload [59, 60]. Privacy standards were considered problematic [89] and lack of private workspaces for virtual consultations further hindered service acceptance and efficiency [95].

### Decreased Quality of Care/Lack of Physical Examinations

Providers viewed telehealth as an insufficient replacement for in-person contact and physical exams [8, 10, 62, 64, 65, 69, 70, 77, 79, 95]. Concerns included the inability to perform physical exams and the psychological burden on providers fearing decreased care quality [58, 62, 68, 69, 88]. Postpartum telehealth faced challenges in assessing critical aspects like mother-infant-bonding, breastfeeding, and recovery [29, 60]. Practical skills training and identifying domestic violence were also less effective virtually [8, 93, 95]. Building trust and managing complex situations were difficult [51, 58, 65], impeding woman-centered care and grief support after stillbirth [32, 76].

## Discussion

This scoping review examined the impact of the COVID-19 pandemic on the working conditions of maternity staff. The data collected provide a comprehensive view of the multifaceted impact, highlighting both the challenges and adaptations experienced by maternity care providers in OECD countries. The eight themes that emerged fell into two broad topics: structural challenges and the impact on mental health among the workforce. Structural challenges included staff shortages, restructuring, inadequate provision of PPE, transition to virtual communication, management of SARS-CoV-2 positive patients, and restrictions on accompanying persons. The impact on mental health was significant, with increased levels of anxiety, stress and moral dilemmas among staff. Despite these challenges, a strong sense of professional solidarity was observed.

Inadequate supply of PPE was a major concern during the COVID-19 pandemic. Staff often had to reuse PPE or faced delays in obtaining essential protective equipment. Inconsistent testing protocols further added to the uncertainty and risk of exposure. Consistent with our findings, two recent Australian studies reported frequent deviations from infection control measures related to the use of PPE by healthcare workers [105, 106]. These findings underscore the need for sufficient provision of PPE to maximize protection, decrease adverse effects, and ensure adherence to proper use. Providing adequate PPE is particularly important for community midwives [80].

The transition to telehealth services was necessary to maintain continuity of care while minimizing the risk of infection. In line with our findings, a recent review shows that, this transition presented challenges, including technical difficulties and reduced interpersonal interactions, which are essential to maternity care [107]. Addressing these barriers through better technical support, clear guidelines, and hybrid models of care could increase the effectiveness of telehealth in obstetric care. Salameh et al. [87] recommend continuous professional education and the adoption of telehealth to enhance quality of care during future health crises.

Our findings align with recent studies indicating that the COVID-19 pandemic significantly impacted the working conditions of healthcare providers across various fields. The pandemic increased workloads, organizational burdens, and created challenges related to staffing, responsibilities, and financial remuneration [3, 108]. Staff shortages often resulted in increased workload and stress for the remaining staff. Consistent with our results, recent studies have shown that these factors negatively impacted the quality of maternal care, as antenatal care visits decreased and the health infrastructure was strained [109, 110]. Additionally, marginalized pregnant and postpartum women appeared to be disproportionately affected by these circumstances [111]. Limiting the presence of partners or support persons during labor and delivery was a controversial issue. While intended to reduce the risk of infection, it often resulted in increased patient anxiety and dissatisfaction, which indirectly affected staff. There is evidence that social containment measures have affected relevant patient outcomes such as breastfeeding [112] or cesarean section rates [6]. Our findings indicate that the COVID-19 pandemic had a significant impact on obstetric procedures, particularly regarding cesarean section rates and the performance of invasive prenatal procedures. A large cohort study from Italy [113] confirms that cesarean section rates initially increased during the pandemic, possibly due to uncertainty in managing infected patients. However, rates declined over time, suggesting that maternity staff gained more confidence in handling COVID-19-positive laboring women. This aligns with our findings, emphasizing the need for continuous adaptation in maternity care to balance infection control measures with evidence-based obstetric practice. Additionally, data from Carbone et al. [114] show a significant reduction in invasive prenatal procedures such as amniocentesis during the pandemic. This decline may have been driven by efforts to minimize hospital visits for non-urgent interventions, as well as concerns about potential infection risks for both mothers and fetuses. These findings highlight the necessity of clear guidelines for performing invasive procedures under pandemic conditions to prevent disruptions in essential prenatal care.

Consistent with our findings, a recent qualitative study among NHS staff revealed that visitor restrictions during the COVID-19 pandemic had mixed effects on maternity care [115]. While these restrictions were essential for infection control and resulted in benefits such as increased job satisfaction and improved communication, they also led to significant emotional challenges and changes in care practices.

As shown in other studies, the COVID-19 pandemic underscored the need for robust organizational structures, effective communication, flexible staffing, and adequate financial support in healthcare [116, 117]. Addressing these areas will enhance preparedness and resilience in future health crises, ensuring the provision of high-quality care for pregnant women and newborns. Ellis et al. emphasized including midwives in community disaster planning and enhancing interprofessional collaboration [45]. Transparent and consistent communication protocols regarding changes in care practices and safety measures can reduce uncertainty and stress among staff and patients. The COVID-19 pandemic further revealed significant gaps in the guidelines and management of maternity care. Addressing these issues through clearer, evidence-based, and unified guidelines can better prepare maternity services for future health crises, ensuring the safety and well-being of mothers, newborns, and healthcare providers.

The mental health impacts on maternity care staff were profound, highlighting the need for better psychological support systems. The pandemic exacerbated existing stressors and introduced new ones, leading to a significant deterioration in mental well-being. Consistent with our findings, a recent synthesis of consensus statements [118] highlights the constant fear of infection among maternity staff, coupled with increased workloads and witnessing the distress of patients and their families. To ensure the well-being of obstetric staff during future health crises, preventive measures and support programs should be established. This includes regular psychological support, stress management workshops and ethical training. Particular attention should be paid to reducing stress factors such as work overload and moral dilemmas in order to minimize the risk of anxiety and depression [34, 40, 43]. Establishing robust mental health support systems, including access to counseling and stress management resources, is essential. Regular mental health check-ins and support groups can help mitigate the psychological toll on staff. The COVID-19 pandemic highlighted the moral obligations and hazards faced by providers of maternity care, challenging their ethical foundations and professional standards. The experience fostered personal growth and professional solidarity, reinforcing the commitment to providing high-quality care despite unprecedented challenges. This period underscored the need for a reflective approach to handling ethical dilemmas in maternity care [119, 120] and health care in general [121], as providers encountered situations that required balancing personal safety with professional duty, often leading to moral distress.

Our scoping review significantly extends the current state of knowledge in several key areas, building upon the foundations laid by Flaherty et al. and Schmitt et al. While Flaherty et al. focused on both women's and providers'perspectives up to early 2021, and Schmitt et al. examined effects on maternity staff in 2020, our review provides a more comprehensive and longitudinal overview of the impact of the COVID-19 pandemic specifically on maternity staff, with studies included up to September 2023. This extended timeframe allowed us to identify long-term trends and adaptation strategies not captured in earlier works, such as the evolution of telehealth practices and the lasting impact on staff mental health. Our analysis delves deeper into structural challenges, revealing nuanced insights into how healthcare systems adapted over time. For instance, we identified the long-term effects of staff redeployment and the gradual development of COVID-19-specific maternity care protocols. Additionally, our review uniquely captures the interplay between structural changes and individual staff experiences, highlighting how organizational adaptations influenced staff well-being and care delivery over an extended period. Moreover, our integration of both qualitative and quantitative data from 83 studies, compared to the 14 studies in Schmitt et al. and the qualitative focus of Flaherty et al., allows for a more comprehensive understanding of the diverse experiences and coping strategies of maternity staff during the pandemic. This mixed-methods approach enabled us to quantify trends while also capturing the rich, lived experiences of healthcare providers. Notably, our review identified emerging themes not prominent in earlier works, such as the development of occupational solidarity among maternity staff and the long-term implications of virtual communication on provider-patient relationships. These findings provide valuable insights for developing resilient maternity care systems and supporting staff in future health crises.

### Strengths and limitations

The present scoping review has several important strengths. Data collection and extraction were performed independently by different authors, and conflicts were discussed until consensus was reached. Studies with different study designs were included. The comprehensive nature of this scoping review, including 83 articles, provides a broad perspective on the impact of COVID-19 on maternity care.

However, we acknowledge several limitations. In our literature search, we only looked for articles from countries belonging to the Organization for Economic Cooperation and Development. By focusing on OECD members, the review minimized confounding variables tied to healthcare disparities, allowing sharper insights into workforce-specific challenges rather than systemic inequities. While this approach strengthened internal validity, we recognize it may have excluded critical perspectives from low-resource settings where pandemic impacts were magnified. Our exclusion criteria precluded insights from low-resource settings where staffing shortages, PPE deficits, and maternal mortality rates were disproportionately severe during the pandemic. For example, sub-Saharan African nations reported midwife-to-population ratios below WHO recommendations even pre-pandemic, a crisis exacerbated by COVID-19. Future reviews should prioritize inclusive geographic scoping to disentangle pandemic-specific stressors from preexisting systemic gaps. While our search included manual screening of German journals, linguistic restrictions may have omitted relevant studies from Spanish-speaking OECD countries. For instance, Chile’s midwife-led"Matronas en Red"network documented unique telehealth challenges in rural areas, yet such reports often remain in regional journals.

The rapidly evolving nature of the pandemic means that some findings may be outdated or not reflective of the current situation. In addition, most studies focused on the immediate impact of the pandemic, with limited data on long-term effects on both maternity care providers and patients.

## Conclusion

Overall, the findings highlight the need for improved support systems for maternity care workers during pandemics to mitigate adverse effects. Recommendations include better resource allocation, improved mental health support, and clear communication strategies to effectively manage future public health crises. The COVID-19 pandemic severely disrupted maternity care services, creating both structural challenges and serious mental health consequences for the workforce. Addressing these issues through improved resource allocation, mental health support, and clear communication strategies is critical to building a resilient maternity care system that can withstand future health crises. Further research is needed to explore the long-term effects of the pandemic on maternity care workers and to develop evidence-based interventions to support their well-being.

## Supplementary Information

Supplementary Material 1. PRISMA-ScR Checklist.

Supplementary Material 2. Search strategy for bibliographic databases used.

Supplementary Material 3. Reports excluded since already referenced in included in reviews.

## Data Availability

Data generated or analyzed during this study are included in this published article (see Table 1 and appendix 2). A detailed dataset generated during the current study, containing detailed data on the extracted results of the included publications, is available from the corresponding author on reasonable request.

## Abbreviations

COVID-19: Coronavirus disease
FT: Full text screening
NHS: National health service (Great Britian)
OECD: Organization for economic cooperation and development
PPE: Personal protective equipment
PRISMA-ScR: Preferred reporting items for systematic reviews and meta-analyses—extension for scoping reviews
SARS-CoV-2: Severe acute respiratory syndrome coronavirus 2
TiAb: Initial title and abstract screening
WHO: World health organization
