# Supplementary material for: Effects of the COVID-19 pandemic on working conditions of maternity staff – a scoping review
Source: BMC Pregnancy Childbirth. 2025 Aug 14;25:855. doi: 10.1186/s12884-025-07905-5 (PMC12355864; doi:10.1186/s12884-025-07905-5)
Supplement: Supplementary file 2 — Supplementary Material 2. Search strategy for bibliographic databases used. [file 12884_2025_7905_MOESM2_ESM.docx]

**Search strategy for bibliographic databases used**

**PUBMED: 971 Records**

**((midwi*[Title/Abstract] OR "nurse-midwi*"[Title/Abstract] OR "Nurse Midwives"[Title/Abstract] OR "obstetric nurs*"[Title/Abstract] OR "perinatal care"[Title/Abstract] OR "maternity care"[Title/Abstract] OR (nurs*[Title/Abstract] AND perin*[Title/Abstract]) OR (nurs*[Title/Abstract] AND matern*[Title/Abstract]) OR obstetric*[Title/Abstract] OR "Perinatal Care"[Title/Abstract] OR "Obstetrics"[Title/Abstract] OR "Obstetricians"[Title/Abstract] OR "Obstetric Nursing"[Title/Abstract]) AND (burden[Title/Abstract] OR workload[Title/Abstract] OR barriers[Title/Abstract] OR challenges[Title/Abstract] OR stress[Title/Abstract] OR "mental health"[Title/Abstract] OR perception*[Title/Abstract] OR narratives[Title/Abstract] OR anxiety[Title/Abstract] OR difficulty[Title/Abstract] OR worry[Title/Abstract] OR accountability[Title/Abstract] OR hindrance[Title/Abstract] OR hurdle[Title/Abstract] OR obstacle[Title/Abstract] OR perspective[Title/Abstract] OR impression[Title/Abstract] OR opinion[Title/Abstract] OR viewpoint[Title/Abstract] OR Workload[Title/Abstract] OR "Mental Health"[Title/Abstract] OR "Health Resources"[Title/Abstract] OR "Narration"[Title/Abstract] OR "Anxiety"[Title/Abstract] OR "Employee Workload"[Title/Abstract] OR "Staff Workload"[Title/Abstract] OR Angst[Title/Abstract] OR Anxiousness[Title/Abstract])) AND (covid[Title/Abstract] OR coronavirus[Title/Abstract] OR "corona virus"[Title/Abstract] OR COVID-19[Title/Abstract] OR Covid2019[Title/Abstract] OR SARS-CoV*[Title/Abstract] OR SARSCov*[Title/Abstract] OR "novel CoV*"[Title/Abstract] OR SARS-CoV-2[Title/Abstract] OR Coronavirus[Title/Abstract] OR "COVID 19"[Title/Abstract] OR "COVID-19 Virus Infection"[Title/Abstract] OR "COVID-19 Pandemic"[Title/Abstract])
Filters: English, German, from 2020/1/1 - 2023/9/25**

**Web of Science: 967 Records**

((AB=(midwi* OR "nurse-midwi*" OR "Nurse Midwives" OR "obstetric nurs*" OR "perinatal care" OR "maternity care" OR (nurs* AND perin*) OR (nurs* AND matern*) OR obstetric* OR "Perinatal Care" OR "Obstetrics" OR "Obstetricians" OR "Obstetric Nursing")) AND AB=(burden OR workload OR barriers OR challenges OR stress OR "mental health" OR perception* OR narratives OR anxiety OR difficulty OR worry OR accountability OR hindrance OR hurdle OR obstacle OR perspective OR impression OR opinion OR viewpoint OR Workload OR "Mental Health" OR "Health Resources" OR "Narration" OR "Anxiety" OR "Employee Workload" OR "Staff Workload" OR Angst OR Anxiousness)) AND AB=(covid OR coronavirus OR "corona virus" OR COVID-19 OR Covid2019 OR SARS-CoV* OR SARSCov* OR "novel CoV*" OR SARS-CoV-2 OR Coronavirus OR "COVID 19" OR "COVID-19 Virus Infection" OR "COVID-19 Pandemic")
**Filters: English, German, from 2020/1/1 - 2023/9/25**

**CINHAHL: 607 Records**

(TI (midwi* OR "nurse-midwi*" OR "Nurse Midwives" OR "obstetric nurs*" OR "perinatal care" OR "maternity care" OR (nurs* AND perin*) OR (nurs* AND matern*) OR obstetric* OR "Perinatal Care" OR "Obstetrics" OR "Obstetricians" OR "Obstetric Nursing") OR AB (midwi* OR "nurse-midwi*" OR "Nurse Midwives" OR "obstetric nurs*" OR "perinatal care" OR "maternity care" OR (nurs* AND perin*) OR (nurs* AND matern*) OR obstetric* OR "Perinatal Care" OR "Obstetrics" OR "Obstetricians" OR "Obstetric Nursing")) AND (TI (burden OR workload OR barriers OR challenges OR stress OR "mental health" OR perception* OR narratives OR anxiety OR difficulty OR worry OR accountability OR hindrance OR hurdle OR obstacle OR perspective OR impression OR opinion OR viewpoint OR Workload OR "Mental Health" OR "Health Resources" OR "Narration" OR "Anxiety" OR "Employee Workload" OR "Staff Workload" OR Angst OR Anxiousness) OR AB (burden OR workload OR barriers OR challenges OR stress OR "mental health" OR perception* OR narratives OR anxiety OR difficulty OR worry OR accountability OR hindrance OR hurdle OR obstacle OR perspective OR impression OR opinion OR viewpoint OR Workload OR "Mental Health" OR "Health Resources" OR "Narration" OR "Anxiety" OR "Employee Workload" OR "Staff Workload" OR Angst OR Anxiousness)) AND (TI (covid OR coronavirus OR "corona virus" OR COVID-19 OR Covid2019 OR SARS-CoV* OR SARSCov* OR "novel CoV*" OR SARS-CoV-2 OR Coronavirus OR "COVID 19" OR "COVID-19 Virus Infection" OR "COVID-19 Pandemic") OR AB (covid OR coronavirus OR "corona virus" OR COVID-19 OR Covid2019 OR SARS-CoV* OR SARSCov* OR "novel CoV*" OR SARS-CoV-2 OR Coronavirus OR "COVID 19" OR "COVID-19 Virus Infection" OR "COVID-19 Pandemic"))
**Filters: English, German, from 2020/1/1 - 2023/9/25**

**COCHRANE Library: 55 Records**

midwi* OR (nurse NEXT midwi*) OR (Nurse NEXT Midwives) OR (obstetric NEXT nurs*) OR (perinatal NEXT care) OR (maternity NEXT care) OR ((nurs) AND (perin*)) OR ((nurs) AND (matern*)) OR obstetric* OR "Perinatal Care" OR "Obstetrics" OR "Obstetricians" OR "Obstetric Nursing" in Title Abstract Keyword AND burden OR workload OR barriers OR challenges OR stress OR "mental health" OR perception* OR narratives OR anxiety OR difficulty OR worry OR accountability OR hindrance OR hurdle OR obstacle OR perspective OR impression OR opinion OR viewpoint OR Workload OR "Mental Health" OR "Health Resources" OR "Narration" OR "Anxiety" OR "Employee Workload" OR "Staff Workload" OR Angst OR Anxiousness in Title Abstract Keyword AND covid OR coronavirus OR "corona virus" OR COVID-19 OR Covid2019 OR SARS-CoV* OR SARSCov* OR (novel Next CoV) OR SARS-CoV-2 OR Coronavirus OR "COVID 19" OR "COVID-19 Virus Infection" OR "COVID-19 Pandemic" in Title Abstract Keyword - with Cochrane Library publication date Between Jan 2020 and Sep 2023, in Cochrane Reviews, Trials (Word variations have been searched)
**Filters: English, German, from 2020/1/1 - 2023/9/25**

**PSYNDEX: 5 Records**

((midwi* or "nurse-midwi*" or "Nurse Midwives" or "obstetric nurs*" or "perinatal care" or "maternity care" or (nurs* and perin*) or (nurs* and matern*) or obstetric* or "Perinatal Care" or "Obstetrics" or "Obstetricians" or "Obstetric Nursing") and (burden or workload or barriers or challenges or stress or "mental health" or perception* or narratives or anxiety or difficulty or worry or accountability or hindrance or hurdle or obstacle or perspective or impression or opinion or viewpoint or Workload or "Mental Health" or "Health Resources" or "Narration" or "Anxiety" or "Employee Workload" or "Staff Workload" or Angst or Anxiousness)).mp. and (covid or coronavirus or "corona virus" or COVID-19 or Covid2019 or SARS-CoV* or SARSCov* or "novel CoV*" or SARS-CoV-2 or Coronavirus or "COVID 19" or "COVID-19 Virus Infection" or "COVID-19 Pandemic").ab,ti. [mp=title, book title, abstract, original title, name of substance word, subject heading word, floating sub-heading word, keyword heading word, organism supplementary concept word, protocol supplementary concept word, rare disease supplementary concept word, unique identifier, synonyms, population supplementary concept word, anatomy supplementary concept word]
**Filters: English, German, from 2020/1/1 - 2023/9/25**

**APA PsychNet: 6 Records**

((midwi* or "nurse-midwi*" or "Nurse Midwives" or "obstetric nurs*" or "perinatal care" or "maternity care" or (nurs* and perin*) or (nurs* and matern*) or obstetric* or "Perinatal Care" or "Obstetrics" or "Obstetricians" or "Obstetric Nursing") and (burden or workload or barriers or challenges or stress or "mental health" or perception* or narratives or anxiety or difficulty or worry or accountability or hindrance or hurdle or obstacle or perspective or impression or opinion or viewpoint or Workload or "Mental Health" or "Health Resources" or "Narration" or "Anxiety" or "Employee Workload" or "Staff Workload" or Angst or Anxiousness)).mp. and (covid or coronavirus or "corona virus" or COVID-19 or Covid2019 or SARS-CoV* or SARSCov* or "novel CoV*" or SARS-CoV-2 or Coronavirus or "COVID 19" or "COVID-19 Virus Infection" or "COVID-19 Pandemic").ab,ti. [mp=title, book title, abstract, original title, name of substance word, subject heading word, floating sub-heading word, keyword heading word, organism supplementary concept word, protocol supplementary concept word, rare disease supplementary concept word, unique identifier, synonyms, population supplementary concept word, anatomy supplementary concept word]
**Filters: English, German, from 2020/1/1 - 2023/9/25**
