# Supplementary material for: Effects of the COVID-19 pandemic on working conditions of maternity staff – a scoping review
Source: BMC Pregnancy Childbirth. 2025 Aug 14;25:855. doi: 10.1186/s12884-025-07905-5 (PMC12355864; doi:10.1186/s12884-025-07905-5)
Supplement: Supplementary file 3 — Supplementary Material 3. Reports excluded since already referenced in included in reviews. [file 12884_2025_7905_MOESM3_ESM.docx]

**Reports excluded as they were already referenced in included in reviews (n = 11)**

Altman, M. R., Gavin, A. R., Eagen-Torkko, M. K., Kantrowitz-Gordon, I., Khosa, R. M., & Mohammed, S. A. (2021). Where the System Failed: The COVID-19 Pandemic's Impact on Pregnancy and Birth Care. Glob Qual Nurs Res, 8, 23333936211006397. https://doi.org/doi:10.1177/23333936211006397 => *referenced in Flaherty et al. 2022*

Baumann, S., Gaucher, L., Bourgueil, Y., Saint-Lary, O., Gautier, S., & Rousseau, A. (2021). Adaptation of independent midwives to the COVID-19 pandemic: A national descriptive survey. *Midwifery*, *94*. https://doi.org/doi:10.1016/j.midw.2020.102918 => *referenced in Schmitt et al. 2021*

Bender, W. R., Srinivas, S., Coutifaris, P., Acker, A., & Hirshberg, A. (2020). The Psychological Experience of Obstetric Patients and Health Care Workers after Implementation of Universal SARS-CoV-2 Testing. *American Journal of Perinatology*, *37*(12), 1271-1279. => *referenced in both Flaherty et al. 2022 and Schmitt et al. 2021*

*Bradfield, Z., Hauck, Y., Homer, C. S. E., Sweet, L., Wilson, A. N., Szabo, R. A., Wynter, K., Vasilevski, V., & Kuliukas, L. (2022). Midwives' experiences of providing maternity care during the COVID-19 pandemic in Australia. Women & Birth, 35(3), 262-271. https://doi.org/doi:10.1016/j.wombi.2021.02.007* => *referenced in Flaherty et al. 2022*

González-Timoneda, A., Hernández Hernández, V., Pardo Moya, S., & Alfaro Blazquez, R. (2021). Experiences and attitudes of midwives during the birth of a pregnant woman with COVID-19 infection: A qualitative study. *Women Birth*, *34*(5), 465-472. https://doi.org/doi:10.1016/j.wombi.2020.12. => *referenced in both Flaherty et al. 2022 and Schmitt et al. 2021*

Kang, H. S., Son, Y., Kim, M. J., & Chae, S. M. (2021). Experiences of nurses caring for perinatal women and newborns during the COVID-19 pandemic: A descriptive qualitative study. *Nursing Open*, *8*(6), 3358-3365. https://doi.org/doi:10.1002/nop2.881 => *referenced in Flaherty et al. 2022*

Khot, N., & Kumar, A. (2020). Flattening the anxiety curve: Obstetricians' response to the COVID-19 pandemic in Victoria. *Aust N Z J Obstet Gynaecol*, *60*(4), E10. https://doi.org/doi:10.1111/ajo.13209 => *referenced in Schmitt et al. 2021*

Madden, N., Emeruwa, U. N., Friedman, A., M., e., Aubey, J. J., Aziz, A., Baptiste, C. D., Coletta, J. M., D'Alton, M. E., Fuchs, K. M., Goffman, D., Gyamfi-Bannerman, C., Kondragunta, S., Krenitsky, N., Miller, R. S., Nhan-Chang, C.-L., Saint Jean, A., M., a., Shukla, H. P., . . . Yates, H. S. (2020). Telehealth Uptake into Prenatal Care and Provider Attitudes during the COVID-19 Pandemic in New York City: A Quantitative and Qualitative Analysis. *American Journal of Perinatology*, *37*(10), 1005-1014. https://doi.org/doi:10.1055/s-0040-1712939 => *referenced in both Flaherty et al. 2022 and Schmitt et al. 2021*

Peahl, A. F., Powell, A., Berlin, H., Smith, R. D., Krans, E., Waljee, J., Dalton, V. K., Heisler, M., & Moniz, M. H. (2021). Patient and provider perspectives of a new prenatal care model introduced in response to the coronavirus disease 2019 pandemic. *American Journal of Obstetrics and Gynecology*, *224*(4). https://doi.org/doi:10.1016/j.ajog.2020.10.008 => *referenced in both Flaherty et al. 2022 and Schmitt et al. 2021*

Szabo, R. A., Wilson, A. N., Homer, C., Vasilevski, V., Sweet, L., Wynter, K., Hauck, Y., Kuliukas, L., & Bradfield, Z. (2021). Covid-19 changes to maternity care: Experiences of Australian doctors. *Australian & New Zealand Journal of Obstetrics & Gynaecology*, *61*(3), 408-415. https://doi.org/doi:10.1111/ajo.13307 => *referenced in Flaherty et al. 2022*

Uzun, N. D., Tekin, M., Sertel, E., & Tuncar, A. (2020). Psychological and social effects of COVID-19 pandemic on obstetrics and gynecology employees. *Journal of Surgery & Medicine (JOSAM)*, *4*(5), 355-358. https://doi.org/doi:10.28982/josam.735384 => *referenced in Schmitt et al. 2021*
